# *Hydroides* Gunnerus, 1768 (Annelida, Serpulidae) is feminine: a nomenclatural checklist of updated names

**DOI:** 10.3897/zookeys.642.10443

**Published:** 2017-01-03

**Authors:** Geoffrey B. Read, Harry A. ten Hove, Yanan Sun, Elena K. Kupriyanova

**Affiliations:** 1 National Institute of Water and Atmospheric Research (NIWA), 301 Evans Bay Parade, Hataitai, Wellington 6021, New Zealand; 2 Naturalis Biodiversity Center, Darwinweg 2, 2333 CR Leiden, the Netherlands; 3 Australian Museum, 1 William Street, Sydney, NSW, 2010, Australia; 4 Department of Biological Science, Macquarie University, Sydney, NSW, Australia

**Keywords:** Etymology, gender agreement, geolocation, ICZN, type locality

## Abstract

As a service to taxonomists and ecologists using names in the well-known and species-rich ship-fouling serpulid genus *Hydroides* we present an update of all 107 non-synonymised scientific names, with additional information on *Hydroides* nomenclature, original names, etymologies, and type localities derived from original literature, and in accord with the World Register of Marine Species (WoRMS) database. An update is needed because the gender of genus *Hydroides* has from 1 January 2000 reverted to the original feminine, due to a change in the wording of International Code of Zoological Nomenclature which was overlooked at that time, and is contrary to the usage in practice of *Hydroides* as masculine which had started about 1992, although Code-required from the 1960s. We match 31 further original names of current WoRMS subjective junior synonyms to each non-synonymised name, and also report on the world distribution of the genus as illustrated by type localities of the valid names. We include notes on seven *species inquirenda*. The correct rendering is given of six names that have been altered for gender agreement for the first time herein. *Hydroides gottfriedi* **nom. n.** replaces junior homonym *Hydroides rostrata* Pillai, 1971. Currently there are 41 non-synonymised species-group names in *Hydroides* which should be gender invariant, and 23 names which would only change if moved to a neuter genus; the remaining 43 names are fully gender variable. Place-names (23), and personal names (16) make up more than a third (36%) of the species names, with most of the remainder (68) being descriptive of species character states, usually of operculum morphology (54). All species, except *Hydroides norvegica* (63°N), have type localities in shallow-water coastal locations in temperate to tropical waters below latitude 44°, with the highest number of new species (54) from the adjoining Western Pacific and Indian Ocean areas. The other concentration of new species (31) are those first found on the Pacific and Atlantic coasts of North America and in the Caribbean.

World Register of Marine Species

## Introduction

An unusual situation has arisen concerning the correct formulation and spelling of historic species-group names in *Hydroides* Gunnerus, 1768 (Serpulidae) with respect to the established requirement of the International Code of Zoological Nomenclature (hereafter the Code) that the suffix spelling of a Latin or Latinized adjectival species-group name must agree in gender with its genus (ICZN 1999: Article 31.2). The stability of *Hydroides* names is important for tracking name usages as it is the largest serpulid genus, currently with 107 non-synonymised names, and also a further 31 original names currently placed as subjective synonyms. *Hydroides* includes *Hydroides elegans* (Haswell, 1883), a model organism for settlement and genetic study (e.g., Hadfield 1998), and some other economically important species such as *Hydroides ezoensis* Okuda, 1934, *Hydroides dianthus* (Verrill, 1873), *Hydroides dirampha* Mörch, 1863, and *Hydroides sanctaecrucis* Krøyer [in] Mörch, 1863, which are foulers of ship hulls, harbour structures, and aquaculture equipment (Sun et al. 2015).

*Hydroides* species are easily recognisable by the morphology of the plug which closes the mouth of their calcareous tubes. It is a two-tier operculum with a basal funnel and a distal spinous structure called the verticil. The distinctive and varied structure of the verticil spines has enabled many *Hydroides* species to be detected. Identification from tubes alone is problematic, thus past diversity is difficult to determine from the fossil record in the absence of the opercula. *Hydroides* has no current subgenera, but *Eupomatus* Philippi 1844, the most prominent junior synonym of *Hydroides*, and the little-used *Eucarphus* Mörch, 1863, were both at times used as subgenera defined by verticil morphology. The taxonomic history is reviewed in Bastida-Zavala and ten Hove (2002).

We recently realised that the gender agreement serpulid taxonomists had been applying to adjectival *Hydroides* species-group names for the last 16 years was the opposite of that required by the fourth edition of the Code (ICZN 1999, effective 1 January 2000), in that authors since that date had continued using or creating masculine instead of feminine Latin forms. Additionally, taxonomists had not consistently observed the different Code rule applicable prior to 1999, with one author producing new *Hydroides* names seemingly of both genders in the same publication (Straughan 1967a). Therefore we have compiled a checklist of *Hydroides* name spellings we believe are currently correct, including also identifying the names not subject to gender agreement.

The derivation of *Hydroides* as an Annelida genus name is known. Stearn (1983: 266) explains that substantives derived from -*oides* were commonly used for new genera before and during Linnæus’ era to indicate resemblance to a genus already known, sometimes just as temporary names. The -*oides* suffix is originally adjectival, transliterated from Greek *οειδης*. Gunnerus had at first used the cnidarian genus of *Hydra* (named for the creature of Greek myth) as the genus name for his new tube-dwelling worm in 1766 correspondence with Linnæus, but shortly thereafter changed it to *Hydroides* for his new species *Hydroides norvegica* as published in 1768, “until Mr. v. Linné makes its genus known” (Gunnerus 1768, Moen 2006). The link to hydrozoans was spurious, but the genus name persisted. Usage of the same spelling applied to true hydrozoans persists in Romance language works, where “hydroïdes” (hydroids) can substitute for the formal higher group name, Hydrozoa. The pair of usages are not homonyms in the strict Code sense, but there is some false positive reporting of the *Hydroides* annelid genus in bioinformatics search results (e.g., from some of the Hydrozoa works of Billard, such as Billard 1907).

*Hydroides* was feminine because Gunnerus clearly treated it as feminine when he used the feminine ‘*norvegica*’ as epithet for the worm instead of the masculine ‘*norvegicus*’ (likewise the calcareous tube was separately named *Serpula norvegica* by Gunnerus). However, in recent years Gunnerus’s original feminine gender assignment for *Hydroides* became not obvious to most. This is exemplified by Moen (2006), who in the summary of her historical paper on Gunnerus reports without qualification that “in 1768 J. E. Gunnerus first described the species *Hydroides norvegicus*”. Although Moen was well aware Gunnerus did not use that spelling she perhaps believed the ‘correct’ masculine ending (although incorrect since 2000) was always to be used, regardless of what was originally written.

The Code in its first edition indicated that genus names ending in -*oides* were to be treated as masculine (ICZN 1961: 33, Article 30(a)(ii) Examples), whereas in botany they were treated as feminine (Stearn 1983: 265). By the time of the Code third edition (ICZN 1985: 30, Article 30(b) Examples) the article text was the same, with the examples text explaining that these masculine genus names were substantivated adjectives, thus for *Hydroides* the adjectival descriptive of ‘hydra-like’ was functioning as a noun. Mandatory gender agreement, although much debated, was retained in the Code fourth edition, but changes were made in order “to simplify the identification of gender in genus-group names” (ICZN 1999: XXVI).

Unexpectedly one of the qualifying clauses now included in the Code fourth edition (ICZN 1999) had a major effect on *Hydroides* Gunnerus by reverting it to feminine status after almost 40 years as the opposite gender. The wording of Article 30.1.4.4 in full is “A compound genus-group name ending in the suffix -*ites*, -*oides*, -*ides*, -*odes*, or -*istes* is to be treated as masculine unless its author, when establishing the name, stated that it had another gender or treated it as such by combining it with an adjectival species-group name in another gender form.” Why the Code editorial group thought the refinement was necessary is unknown, but presumably it was regarded as better matching contemporary practice with the original 18–19^th^ century usages.

*Hydroides* began as feminine in 1768, and feminine adjectival endings matching this were usual for over 220 years but not universal (e.g., *Hydroides bifurcatus* Pixell, 1913). Hartman (1965: 79) had maintained original feminine endings in her supplementary world catalogue, although not long later she had used the masculine for *Hydroides pacificus* Hartman (Hartman 1969). Masculine endings, which the Code had required from the early 1960s onwards, otherwise only became common around 1992 (Moen 2006: 121), although *Hydroides bifidus* Imajima, 1982 and *Hydroides bisectus* Imajima & ten Hove, 1989 were newly described somewhat earlier. Ben-Eliahu and ten Hove (1992: 37) correctly pointed out that the Code third edition (ICZN 1985) had *Hydroides* as masculine (actually in place since the first edition). Serpulid taxonomists then adopted the use of masculine endings and continued with this right up to August 2015, unaware of the change back to feminine required from 1 January 2000 when the new Code came into effect. The fourth edition Code was incorrectly cited as continuing masculine endings in ten Hove and Ben-Eliahu (2005: 128). In summary, for nearly 40 years species names in *Hydroides* were required to have masculine endings according to the ICZN Code, although largely ignored for about 30 years, and now for the last 16 years they were required to have the feminine ending as begun by Gunnerus, also ignored. The conflict was first reported in WoRMS by one of us (GBR) in July 2015, after a misinterpretation of the Code requirement for *Hydroides* names was published in Tovar-Hernández et al. (2016, as first online July 2015, p.8). Gender-corrected names were subsequently used in Sun et al. (2015), Kupriyanova et al. (2015), and Sun et al. (2016).

Code Article 31 (ICZN 1999) explains some of the requirements and exceptions regarding species-group Latin name formation. In general, most species-group names ending in the suffixes -*us*, -*a*, -*um* are declinable and likely to be adjectives. There are some exceptions applicable here such as -*spina*, which is a noun in apposition and should not change with gender, and most other name endings will not change. The only possible endings of changeable adjectives are -a, -us (these two make up over 60% of all names), -is, -um, -e, -er, -ior, whereas nouns can have all endings (Welter-Schultes 2013). Personal names as species-group names are usually (exceptions) formed as genitive-case nouns (ICZN 1999, Article 31.1). Nouns with Latin adjectival suffixes can become adjectives, notably non-Latin place-names with the suffixes -*ensis* (masculine/feminine) or -*ense* (neuter), indicating of that place, or suffix ‘-*anus* -*a*’, indicating belonging to. However, nouns compounded with dictionary Latin adjectives are treated as noun phrases in apposition (ICZN 1999, glossary).

Here we present an update of all non-synonymised names, and additional information on *Hydroides* nomenclature, as derived in conjunction with the World Register of Marine Species (WoRMS) Polychaeta database (Read and Fauchald 2016), where further details are available. Certain *species inquirenda* (seven names which are otherwise valid but require taxonomic clarification due to inadequate original descriptions) are included and examined in the checklist for analytic purposes, while noting (as explained by ICZN 1999, Article 23.9.6) that the inclusion of these names should not later be considered as new usages.

We have taken this opportunity to investigate type localities of all the species, and to geolocate them to modern standards if possible. Prior to satellite-based navigation only vessel-based collections were likely to provide type-locality geolocations, and the descriptions used to pinpoint coastal sites could be vague or problematic. For instance Treadwell (1939: 164) gave an update that the “precise position” of his Mayagüez Bay station 6062 of 1902 could be relocated based on using a red buoy at the harbour entrance as a reference point, but we are doubtful of the 100 year longevity of this buoy. We have been able to suggest placements for at least three species for which only a vague location was previously available. Type localities are mapped to show the world distribution.

## Methods

The checklist is based on a review of original literature for all *Hydroides* species-group names, and a review of about 250 *Hydroides*-related name records at WoRMS (Read and ten Hove 2016). Under Code Article 34.2 (ICZN 1999), prior usages in literature are not required—here we simply formulate and present correct spellings. Gender agreement is mandatory, which means that non-agreeing scientific names strictly do not exist as valid spellings, and can be updated without explanation (to the bewilderment of many in the past, so we strongly advise annotation of new gender-spelling variants to avoid uncertainty). It is also worth noting that, while gender-agreement variants obviously are minor spelling changes, the Code is worded so that these different spellings are not treated as separate usages under prevailing usage rules.

All original literature for *Hydroides* names was examined. Names as given are our derivations of correct endings for gender agreement, and are followed as necessary with the original binominal combination and comments on current status. The etymology (author’s and/or interpreted dictionary entries) is given, followed by our evaluation of the type of name (adjective, invariant noun in apposition, etc.) from available information. The derivation of names is unambiguous when authors give full etymologies, but this is rare for old names, and often sketchy for modern ones. Derivations are frequently only evident by matching likely character states mentioned, and occasionally there seems no obvious basis for the name chosen. The sources we have used to study derivations include online dictionaries and meta Greek/Latin language resources (Harper’s Etymonline; Logeion; Lexilogos), the Lewis and Short (1891) Latin dictionary (print, also online), as well as analytical dictionaries on the classical languages in science (Brown 1956; Stearn 1983). We have included the current subjective synonyms at WoRMS (if any) of each name (and their type localities), but have not included the other superseded recombinations, nor any misspellings of the valid name (these are fully listed at WoRMS and links to the current and original name records at WoRMS are in the Suppl. material 1 which also summarises the name analysis).

The type locality names have been investigated and their geolocations are included, usually derived by retrospective georeferencing. They are mapped to place the original discoveries in a geographic context and to locate where topotype material could be sought. Current place-names were geolocated using several web-based gazetteers (e.g., GeoNames, GEOLocate, Marineregions (WoRMS), Wikipedia). Disused historic names were sought via general web searches and Wikipedia. Holotype georeference information in online collections databases and in subsequent publications was evaluated if available (these data can be based on retrospective approximations, rather than information supplied by authors on labels). Occasionally modern authors have published geolocations that are obviously imprecise or displaced, and we have pointed these out. The point-geolocations of the older taxa are our informed coastal assignments (indicated as map estimates) if derived from place-names which are towns, islands, or occasionally only known as strips of coast or other imprecise geographic extents. Sometimes positioning was assisted by further information from or about authors. A few times we were unable to narrow the collection site to any point and we indicate when we have given a general geolocation instead. We are unable to calculate the uncertainties (in extent) of our derived coordinates, and caution that each is a precise point location of the possible site, the nearest logical coastal geolocation at this time, as adjusted with satellite image overlay of terrain using the Wikipedia GeoLocator mapping tool. A list of geolocations is in the Suppl. material 1.

## Results

### Checklist of *Hydroides* species original names

**Family SERPULIDAE Rafinesque, 1815**

***Hydroides* Gunnerus, 1768**

Type species. *Hydroides norvegica* Gunnerus, 1768 (original binomen)

Includes *Eupomatus* Philippi, 1844, type species *Eupomatus uncinatus* Philippi, 1844 (by subsequent designation), *Eucarphus* Mörch, 1863 (as Hydroides (Eucarphus)), type species uncertain (full synonymy in WoRMS)

***Hydroides adamaformis* Pillai, 2009** (original binomen)

Etymology: The author states the name for *Hydroides adamaformis* is derived from Latin *adamas* ‘diamond’ in reference to the diamond-shaped appearance of the verticil spines “although their distal ends are curved inwards”. The suffix -*formis* ‘shaped’ is used to form an adjective.

Evaluation: Masculine/feminine invariant adjective (*formis*, neuter *forme*) (Stearn 1983: 94).

Type locality: Lucas Island (south west corner), near Dampier Archipelago, Kimberley region, Western Australia.

Geolocation: -15.2167°, 124.5167° (author, but is east of Lucas).

WoRMS: 555194

Synonyms: No subjective synonyms.

***Hydroides affinis* (Marion, 1875) (originally as *Eupomatus affinis*)**

Status: Name now disused and representing a *species inquirenda* possibly senior to *Hydroides helmata* (Iroso, 1921).

Etymology: Not stated, but *Eupomatus affinis* is named from the Latin adjective *affinis* ‘related to’.

Evaluation: Masculine/feminine invariant adjective (*affinis* -*e*) (Stearn 1983: 94).

Type locality: Golfe de Marseille, France, Mediterranean Sea. No further precision, but likely to be coastal close to Marseille, possibly at or near Île Ratonneau, which the author mentions frequently (Marion and Bobretzky 1875). However, a stone pier off Arenc, Marseille is also mentioned (as *Hydroides uncinata* habitat).

Geolocation: 43.2872°, 5.3143° (map estimate, Île Ratonneau).

WoRMS: 383237

Synonyms: As *species inquirenda* has no synonyms although *Hydroides helmata* has been suggested (Zibrowius 1971: 713–714).

***Hydroides alatalateralis* (Jones, 1962) (originally as *Eupomatus alatalateralis*)**

Etymology: The author states that *Eupomatus alatalateralis* is named for “limbations that are to be found on the sides of the spines of the distal opercular circlet”, thus combining the Latin adjectives *alata* ‘furnished with wings’ and *lateralis* ‘lateral’.

Evaluation: Masculine/feminine invariant adjective (*lateralis* -*e*) (Stearn 1983: 94).

Type locality: Port Royal, Jamaica, Caribbean Sea.

Geolocation: 17.9369°, -76.8439° (map estimate).

WoRMS: 369228

Synonyms: No subjective synonyms.

***Hydroides albiceps* (Grube, 1870) (originally as Serpula (Eupomatus) albiceps)**

Etymology: Not stated, but *Serpula albiceps* may be named for the *operculum album* mentioned by Grube (1870: 521) by combining the Latin adjective *albus* ‘white’ with -*ceps* derived from Latin noun *caput* ‘head’.

Evaluation: Noun in apposition (cf. noun ‘*quadriceps*’), or if treated as adjectival -*ceps* endings are invariant.

Type locality: ‘Tor’ (El Tor), Gulf of Suez, Red Sea.

Geolocation: 28.2365°, 33.6130° (map estimate).

WoRMS: 130997

Synonyms: *Hydroides spiratubus* Pillai, 2009 (Fenelon Island, Kimberley, Australia)

Serpula (Hydroides) multispinosa ternatensis Fischli, 1903 (Ternate, Indonesia)

***Hydroides amri* Sun, Wong, ten Hove, Hutchings, Williamson & Kupriyanova, 2015 (original binomen)**

Etymology: The authors state the name for *Hydroides amri* is in honour of the Australian Museum Research Institute (AMRI).

Evaluation: Invariant non-Latinized noun in apposition ‘*amri*’ from an acronym, pronounced as a single word, not letter by letter as if an initialism (ICZN 1999, Article 11.3).

Type locality: Bass Point south, south of Wollongong, NSW, Australia.

Geolocation: -34.6033°, 150.8953° (authors).

WoRMS: 852781

Synonyms: No subjective synonyms.

***Hydroides ancorispina* Pillai, 1971 (original binomen)**

Etymology: Not stated, but *Hydroides ancorispina* may be named from Latin nouns *ancora* ‘anchor’, *spina* ‘thorn’, referring to the fact that both radii and verticil spines have anchor shaped tips.

Evaluation: Invariant noun in apposition.

Type locality: Wellawate, Colombo, Sri Lanka.

Geolocation: 6.8746°, 79.8569° (map estimate).

WoRMS: 328434

Synonyms: No subjective synonyms.

***Hydroides arnoldi* Augener, 1918 (original binomen)**

Etymology: Not stated, but *Hydroides arnoldi* is evidently named after one of its collectors, Arnold Schultze.

Evaluation: Invariant genitive noun *arnoldi* from modern personal name of Arnold.

Type locality: Reported as two worms from two collection sites, Lome, Togo and Isla Annobón, Equatorial Guinea, both in Gulf of Guinea, West Africa. However, only the Annobón occurrence remains in *Hydroides arnoldi* (see WoRMS for further explanation).

Geolocation: -1.4063°, 5.6373° (Annobón, map estimate).

WoRMS: 338000

Synonyms: No subjective synonyms.

***Hydroides augeneri* Zibrowius, 1973 (original binomen)**

Etymology: Not stated, but *Hydroides augeneri* is evidently named after Hermann Augener.

Evaluation: Invariant genitive noun *augeneri* from modern personal name of Augener.

Type locality: “Malembe” in Zaire (now Democratic Republic of Congo), but a coastal instance of the name could not be found, either in DR Congo or its neighbours. Zaire (DR Congo) has a very narrow access to the coast, and a coastal georeference was derived from “Vista”, the only other named collection site.

Geolocation: -5.8763°, 12.283° (map estimate for Vista).

WoRMS: 328435

Synonyms: No subjective synonyms.

***Hydroides azorica* Zibrowius, 1972b (original binomen)**

Etymology: Not stated, but evidently *Hydroides azorica* is named after the Azores archipelago where collected.

Evaluation: Latinized adjectival form *azorica* with correct feminine ending. Hydroides ‘azoricus’ usages exist (e.g., Bellan 2001).

Type locality: On shipwreck “Doria” east of Ponta Delgada port, Ilha de Sao Miguel, Açores (Azores).

Geolocation: 37.7410°, -25.6478° (map estimate).

WoRMS: 328436

Synonyms: No subjective synonyms.

***Hydroides bandaensis* Zibrowius, 1972a (original binomen)**

Etymology: Not stated, but evidently *Hydroides bandaensis* is named after the Banda Islands where collected.

Evaluation: Masculine/feminine invariant ‘-*ensis*’ adjective created from non-Latin geographic name Banda.

Type locality: Banda Islands (exact location unknown), Banda Sea, Indonesia.

Geolocation: -4.525°, 129.9089° (gazetteer, for Banda Islands).

WoRMS: 369229

Synonyms: No subjective synonyms.

***Hydroides bannerorum* Bailey-Brock, 1991 (original binomen)**

Etymology: The author named *Hydroides bannerorum* after biologists Albert H. (Hank) and Dora May (Dee) Banner.

Evaluation: Invariant plural genitive adjective *bannerorum* from Banner family name.

Type locality: Near Banners Point (Kalaeloa) sewage outfall, near Pearl Harbour, Oahu, Hawaii, Pacific Ocean.

Geolocation: 21.2719°, -158.1213° (map estimate).

WoRMS: 328437

Synonyms: No subjective synonyms.

***Hydroides basispinosa* Straughan, 1967a (originally as *Hydroides basispinosus*)**

Status: The synonymy of *Hydroides basispinosa* and *Hydroides gradata* Straughan, 1967a with *Hydroides operculata* Treadwell, 1929 was re-confirmed by Sun et al. (2015: 63), but is being re-examined, and we provisionally include the *Hydroides basispinosus* original name analysis separately.

Etymology: Not stated, but the compound name for *Hydroides basispinosus* means ‘spiny-pedestal’ as derived from Latin (originally Greek) feminine noun *basis* ‘pedestal’ and adjective *spinosus* -*a* -*um* ‘spiny’. Basal internal spinules on opercular spines are mentioned (not figured).

Evaluation: Gender-variable adjective (in practice). Elsewhere in the article Straughan used feminine adjectival new species names, and in relation to the basal spinules Straughan probably intended another adjectival compound name. Her error in gender ending can be corrected to ‘-*spinosa*’. However, she used the feminine Latin noun ‘*basis*’ (pedestal), not the adjectival ‘*basalis*’ (basal) which would have become ‘*basalispinosa*’. If a noun phrase with a feminine noun then ‘*basispinosus*’ was incorrect Latin (it should also have been ‘*basispinosa*’), and the original spelling must be maintained (ICZN 1999, Article 31.2.1). While this can be noted, Straughan is not the only author to adopt ‘*basis*’ as if adjectival, and it seems best not to apply the strictest interpretation here. Usage as ‘*basispinosa*’ already exists (e.g., Sun et al. 2015: 63).

Type locality: Mouth of Ross River, Townsville, Queensland, Australia.

Geolocation: -19.2569°, 146.8494° (map estimate).

WoRMS: 881640

Synonyms: See *Hydroides operculata* comments.

***Hydroides bifurcata* Pixell, 1913 (originally as *Hydroides bifurcatus*)**

Etymology: Not stated, but the name for *Hydroides bifurcata* is adjectival from Latin *furcatus* ‘forked’, likely referring to the bifid verticil spines.

Evaluation: Gender-variable adjective. The original incorrect masculine ending as *Hydroides bifurcatus*, repeated in Day (1951: 64), was silently corrected to ‘*bifurcata*’ in Day (1967: 808).

Type locality: Minicoy/Maliku (as Minikoi), south Lakshadweep archipelago, north of the Maldive Islands.

Geolocation: 8.2854°, 73.0673° (map estimate).

WoRMS: 873900

Synonyms: No subjective synonyms.

***Hydroides bisecta* Imajima & ten Hove, 1989 (originally as *Hydroides bisectus*)**

Etymology: Not stated, but *Hydroides bisecta* is likely named based on *bisectus* ‘bisected’, a New Latin past participle used as an adjective, derived from Latin *bis* ‘two’, *secare* ‘to cut’, and referring to the bifid tips of verticil spines.

Evaluation: Gender-variable adjective, corrected herein to ‘*bisecta*’.

Type locality: off Sesoko Marine Station, Sesoko Island, Okinawa Islands, Japan.

Geolocation: 26.6365°, 127.8661° (map estimate).

WoRMS: 880526

Synonyms: No subjective synonyms.

***Hydroides bispinosa* Bush, 1910 (original binomen)**

Etymology: Not stated, but the name for *Hydroides bispinosa* is likely referring to the pair of lateral spinules on the verticil spines described by Bush, based on Latin *bis* ‘two’ with adjective *spinosus* ‘spined’. Bush compared *Hydroides bispinosa* with *Hydroides multispinosa*.

Evaluation: Gender-variable adjective with correct original feminine ending. Usage as ‘*bispinosus*’ exists (e.g., Bastida-Zavala and ten Hove 2002: 125).

Type locality: Bermuda. The Yale Peabody Museum type (syntype? YPM IZ 001367.AN) from the Verrill Bermuda Expedition in 1898, evidently has no further location data, but Castle Harbour is a collection site mentioned by Bush (1910).

Geolocation: Imprecisely known (possible place of origin, Castle Harbour, 32.3472°, -64.6872°, Bermuda).

WoRMS: 421083

Synonyms: No subjective synonyms.

***Hydroides brachyacantha* Rioja, 1941a (original binomen)**

Etymology: Not stated, and the description of *Hydroides brachyacantha* does not indicate why the name derives from Greek βραχυ (brachy) ‘short’, ακανθα (akantha) ‘spine’, feminine noun, thus short-spine. In New Latin *acantha* has frequently been used as part of feminine compound names in both genera and species-group names. An identical spelling might be expected to be a noun form in both, but species-group names ending as -*acantha* -*acanthus* have regularly been treated as Latinized Greek adjectives, and that may have been the intention of the author.

Evaluation: Gender-variable adjective with correct feminine ending. Usages as ‘*brachyacanthus*’ exist (e.g., Bastida-Zavala and ten Hove 2003: 73).

Type locality: Marina Mazatlán, Mazatlán, Sinaloa, Gulf of California, Mexico.

Geolocation: 23.2797°, -106.4611° (original author, with neotype of Sun et al. (2016: 49) from the same geolocation).

WoRMS: 328441

Synonyms: No subjective synonyms.

***Hydroides bulbosa* ten Hove, 1990 (originally as *Hydroides bulbosus*)**

Etymology: Not stated, but *Hydroides bulbosa* is evidently named for the bulbous (Latin *bulbosus* -*a* -*um*) dorsal verticil spine.

Evaluation: Gender-variable adjective, corrected herein to ‘*bulbosa*’.

Type locality: Khor Ghubb ’Ali, Musandam Peninsula, Oman, Strait of Hormuz, in a sheltered bay at 18 m.

Geolocation: 26.2633°, 56.3572° (map estimate).

WoRMS: 882354

Synonyms: No subjective synonyms.

***Hydroides calopoma* Zibrowius, 1973 (original binomen)**

Etymology: Not stated, but the name for *Hydroides calopoma* is a compound noun which may be referring to the operculum, from Greek καλος (kalos) ‘beautiful’, πωμα (poma) ‘lid’.

Evaluation: Invariant noun in apposition (indeclinable because ending in a transliterated Greek word).

Type locality: Isla Tortuga, off Isla Annobón, Equatorial Guinea, Gulf of Guinea.

Geolocation: -1.4055°, 5.6562° (map estimate).

WoRMS: 369230

Synonyms: No subjective synonyms.

***Hydroides capensis* Zibrowius, 1972b (original binomen)**

Etymology: Not stated, but *Hydroides capensis* is evidently named after the Cape Provinces of South Africa.

Evaluation: Masculine/feminine invariant ‘-*ensis*’ adjective created from non-Latin geographic name.

Type locality: Offshore from Lambert’s Bay, north of Cape Town, western coast of South Africa.

Geolocation: -32.0833°, 17.9333° (author).

WoRMS: 338003

Synonyms: No subjective synonyms.

***Hydroides chilensis* Hartmann-Schröder, 1962 (original binomen)**

Etymology: Not stated, but *Hydroides chilensis* is evidently named after the country of collection.

Evaluation: Masculine/feminine invariant ‘-*ensis*’ adjective created from non-Latin geographic name.

Type locality: Arica (coastal port city), Chile.

Geolocation: -18.4815°, -70.3333° (map estimate).

WoRMS: 328444

Synonyms: No subjective synonyms.

***Hydroides crucigera* Mörch, 1863 (originally as Hydroides (Eucarphus) crucigera)**

Etymology: Not stated, but the name for *Hydroides crucigera* is likely referring to the verticil spines, which are cross-bearing, from feminine Latin noun *crux*, *crucis* ‘cross’, with Latin suffix *ger*, *gera* ‘to bear’.

Evaluation: Gender-variable adjective with correct original feminine ending. Usages as incorrect suffix ‘*crucigerus*’ and as masculine ‘*cruciger*’ exist (e.g., de León González 1990: 336, Bastida-Zavala and ten Hove 2003: 78). Names ending in -*ger* may be nouns or masculine adjectives (ICZN 1999, Article 31.2.2). The usage of Mörch was adjectival as he used feminine -*gera*.

Type locality: Puntarenas, Gulf of Nicoya, Costa Rica Pacific coast (Mörch 1863 as “*oceano pacifico*, *juxta* Puntarenas”).

Geolocation: 9.9739°, -84.8330° (map estimate).

WoRMS: 333637

Synonyms: *Hydroides californicus* [sic] Treadwell, 1929 (“Lower California” (Baja California) Mexico)

***Hydroides dafnii* (Amoureux, Rullier & Fishelson, 1978) (originally as *Eupomatus dafnii*)**

Etymology: Not stated, but *Eupomatus dafnii* as “trouvé par Mr. Dafni” is evidently named after the collector, Yaacob Dafni (Amoureux et al. 1978: 60, 148).

Evaluation: Invariant genitive form *dafnii* of the modern personal name Dafni.

Type locality: Eilat, Gulf of Aqaba (of Eilat), Israel, Red Sea, on reef coral. The site is mapped by the authors, but not georeferenced.

Geolocation: 29.5266°, 34.9377° (map estimate).

WoRMS: 369231

Synonyms: No subjective synonyms.

***Hydroides deleoni* Bastida-Zavala & ten Hove, 2003 (original binomen)**

Etymology: The authors state *Hydroides deleoni* is named after Jesús A. de León-González.

Evaluation: Invariant genitive form *deleoni* constructed from the personal name de Leon.

Type locality: Punta San Juanico, Western coast of Baja California Sur, Mexico. Authors’ georeference (26°13'N, 112°13'W, inland, ~26 km off target) is herein corrected to 26°15'9"N, 112°28'33"W).

Geolocation: 26.2524°, -112.4757° (San Juanico, map estimate).

WoRMS: 328445

Synonyms: No subjective synonyms.

***Hydroides dianthus* (Verrill, 1873) (originally as *Serpula dianthus*)**

Etymology: Verrill (1873: 28) states for *Serpula dianthus* that the name alludes to the resemblance to *Dianthus* flowers as the colours of its branchiae “recalls the varied hues and forms of different kinds of pinks, (*Dianthus*.)”. The botanical generic name *Dianthus* (flower of Zeus) is New Latin (Linnæus and earlier) from Greek Δηοσ (Dios), genitive of Zeus, and ανθος (anthos) ‘flower’. As *Serpula* is feminine and *dianthus* is masculine it seems Verrill intended the name as a noun (*Actinia dianthus* Ellis, 1768 is an earlier similar pairing).

Evaluation: Invariant noun in apposition.

Type locality: Great Egg Harbor to New Haven and Cape Cod, Atlantic coast USA.

Geolocation: Unknown (New Haven, 41.2520°, -72.9086°, as a central possible place of origin on Atlantic coast USA).

WoRMS: 131000

Synonyms: Possibly *Hydroides hexagonus* *sensu* Pratt, 1916 and others [*non* Bosc, 1802]

*Serpula dianthus citrina* Verrill, 1873 (for Verrill’s colour variant specimens)

Hydroides (Eupomatus) dianthoides Augener, 1922 [*partim*, *fide* Bastida-Zavala and ten Hove 2002: 143) (Haiti, Caribbean Sea)

***Hydroides diplochone* (Grube, 1878a) (originally as Serpula (Hydroides) diplochone)**

Status: Name now disused and representing a *species inquirenda*. A single subsequent valid usage of the name was later identified as an occurrence of *Hydroides ezoensis* (a junior name), but it is uncertain that Grube’s original serpulid (type missing) was the same (*fide* Zibrowius 1978: 144; Sun et al. 2015: 37).

Etymology: Not stated, but the name for *Serpula diplochone* derives from Greek Latinized as *diplos* ‘two-fold’ and feminine Greek noun χοάνη (choani) ‘funnel’, thus double funnel, evidently in reference to the two-tier operculum that Grube describes (a generic character). There are no other names based on *chone* in Serpulidae, but it is part of several generic names in Sabellidae.

Evaluation: Invariant noun in apposition.

Type locality: Askold Island, outer Peter the Great Gulf, North Japan Sea. We infer this to be the type locality. Grube does not present location information beyond that the material was from “nordjapanischen Meeres”, but it is also mentioned that the collector was the Siberian-based Polish naturalist Dybowski, whose travels in the region are documented. In 1874 Benedykt Dybowski collected fauna at Askold Island, near Vladivostok, Primorsky Krai (*fide* Zoological Museum, University of Lliv [no date]).

Geolocation: 42.7333°, 132.3333° (map estimate, Askold Island).

WoRMS: 333639

Synonyms: As *species inquirenda* has no synonyms, although *Hydroides ezoensis* has been suggested.

***Hydroides dipoma* (Schmarda, 1861) (originally as *Eupomatus dipoma*)**

Etymology: Not stated, but in the description for *Eupomatus dipoma* Schmarda (1861: 29) describes in Latin “*Operculum duplex infundibuliforme*” (double funnel lid) and in German “Das Thier hat zwei Deckel” (has two lids), evidently referring to the two opercula figured in his plate, and based on Greek δις (dis) ‘twice’, and πωμα (poma) ‘cap’ (see ten Hove and Ben-Eliahu (2005) for an analysis of records of bi-operculate specimens in *Hydroides*).

Evaluation: Invariant compound noun in apposition (indeclinable because ending in a transliterated Greek word).

Type locality: Cape of Good Hope, South Africa (“Vorgebirge der Guten Hoffnung”).

Geolocation: -34.3583°, 18.4725° (Cape of Good Hope (gazetteer), although Schmarda more likely was indicating a general coastal area).

WoRMS: 369232

Synonyms: *Eupomatus spinosus* Pixell, 1913 (Gulf of Suez)

*Hydroides uncinatus macronyx* Ehlers, 1913 (Simonstown, False Bay, South Africa)

***Hydroides dirampha* Mörch, 1863 (originally as Hydroides (Eucarphus) dirampha)**

Etymology: Not stated, but for *Hydroides dirampha* Mörch describes “*utrinque inflexione obsoleta, unde lateraliter adunco-rostrato*” (rudimentary bend both sides, hence laterally-curved beak), with the Latinization *dirampha* evidently referring to the twin sharp lateral points of the blunt tip of the verticil spines, ultimately from Greek δις (dis) ‘twice’, and ραμφος (rampos) ‘beak’, the latter modified through New Latin masculine noun forms *rhamphus* and the lesser-used *ramphus* (both with a number of usages as part of compound genus names) to *rampha*, a usage seemingly unique to Mörch.

Evaluation: Treated here as an invariant noun in apposition, because an incorrect Latinization. While Mörch consistently modified his *Hydroides* names as feminine, and the name seems intended as feminine adjectival rather than a noun, it looks like a misspelled Latinization which should be left unaltered. Usages in *Hydroides* as ‘*diramphus*’ exist (e.g., Bastida-Zavala and ten Hove 2002: 161).

Type locality: Saint (St.) Thomas Island, United States Virgin Islands, Lesser Antilles (“*in portu urbis St. Thomae Antillarum*”), most likely the Saint Thomas port town of Charlotte Amalie.

Geolocation: Imprecisely known (near to 18.34°, -64.92° if harbour at Charlotte Amalie, St Thomas Island).

WoRMS: 131001

Synonyms: *Eucarphus serratus* Bush, 1910 (Bermuda, western Atlantic)

*Eupomatus lunulifer* Claparède, 1870a (Gulf of Naples, Italy, Tyrrhenian Sea)

Hydroides (Eucarphus) benzoni Mörch, 1863 (Bahia coast, Brazil)

Hydroides (Eucarphus) cumingii Mörch, 1863 (Philippines unspecified)

Hydroides (Eucarphus) cumingii navalis Mörch, 1863 (New Zealand unspecified)

*Hydroides malleophorus* [sic] Rioja, 1942 (Mazatlán, Gulf of California, Mexico Pacific coast)

***Hydroides dolabrus* Tovar-Hernández, Villalobos-Guerrero, Kupriyanova & Sun, 2016 (original binomen)**

Etymology: The authors state for *Hydroides dolabrus* that “*dolabrus* is from the Latin *dolabra*, a sort of pickaxe that resembles the shape of the verticil spines”.

Evaluation: Invariant noun in apposition. The Latin *dolabra* is a feminine noun, and cannot become a masculinised adjective as ‘*dolabrus*’ to match a masculine *Hydroides*. This is not a word as listed in classical Latin dictionaries, and should be considered as an invariant combination of letters. A suitable adjectival equivalent would have been *dolabratus* -*ata*.

Type locality: Mazatlan Marina, Mazatlan, Gulf of California, Mexico Pacific coast.

Geolocation: 23.2798°, -106.4611° (authors).

WoRMS: 851651

Synonyms: No subjective synonyms.

***Hydroides elegans* (Haswell, 1883) (originally as *Eupomatus elegans*)**

Status: The much-used name *Hydroides elegans* is *nomen protectum* with respect to *nomen oblitum H. abbreviata* Krøyer [in] Mörch, 1863 (Bastida-Zavala and ten Hove 2002).

Etymology: Not stated, but the *Eupomatus elegans* name is likely derived from the Latin adjective *elegans* -*antis* (genitive) ‘elegant’.

Evaluation: Invariant adjective (masculine/feminine/neuter ‘-*ans*’).

Type locality: Port Jackson, NSW, Australia (not further specified).

Geolocation: -33.8456°, 151.2622° (gazetteer).

WoRMS: 131002

Synonyms: *Hydroides abbreviata* Krøyer [in] Mörch, 1863 [*nomen oblitum*] (Saint Croix island, Virgin Islands, Caribbean Sea)

*Hydroides incrustans* Monro, 1938 (Shoreham Harbour Canal, Sussex, England)

*Hydroides pacificus* Hartman, 1969 (Velero station 1452-42, Ship hull & pier, Long Beach, California)

*Hydroides spinalateralis* Straughan, 1967a (Shoal Point, Mackay, Queensland, Australia)

***Hydroides elegantula* (Bush, 1910) (originally as *Eupomatus elegantulus*)**

Etymology: Not stated, but the *Eupomatus elegantulus* name is likely derived from Latin adjective *elegans* -*antis* ‘elegant’, combined with the Latin suffix -*ulus*, a male diminutive adjectival form.

Evaluation: Gender-variable adjective, corrected in *Hydroides* to feminine ‘*elegantula*’ by Zibrowius (1971: 695).

Type locality: Bermuda. The Yale Peabody Museum holotype YPM IZ 001323.AN from the Verrill Bermuda Expedition in 1898, evidently has no further location data, but Castle Harbour is a collection site mentioned by Bush (1910).

Geolocation: Imprecisely known (possible place of origin, Castle Harbour, 32.3472°, -64.6872°, Bermuda).

WoRMS: 873929

Synonyms: No subjective synonyms.

***Hydroides euplaeana* (Delle Chiaje, 1828) (originally as *Sabella euplaeana*)**

Status: Name now disused and representing a *species inquirenda* that has been compared to *Hydroides pseudouncinata* Zibrowius, 1968. It is not a candidate *nomen oblitum* (used as valid by Zibrowius 1972c: 116–117). If suppression is desirable prevailing usage of *Hydroides pseudouncinata* would be maintained (ICZN 1999, Recommendation 23A).

Etymology: Not stated, but *Sabella euplaeana* was evidently named after the Latin name for Caiola Island, Naples, where Delle Chiaje states it was collected. Caiola is modern day Gaiola, in Roman times known as Euplaea. The name as combined with feminine adjectival suffix -*ana* indicates from Euplaea.

Evaluation: Gender-variable geographical Latin adjective (-*anus*, -*ana*) from place-name, with correct original feminine ending.

Type locality: Caiola (Gaiola/Euplaea) Island, Naples, Italy, Tyrrhenian Sea, Mediterranean.

Geolocation: 40.7917°, 14.1869° (map estimate).

WoRMS: 381073

Synonyms: As *species inquirenda* has no synonyms, although *Hydroides pseudouncinata* has been suggested.

***Hydroides exaltata* (Marenzeller, 1885) (originally as *Eupomatus exaltatus*)**

Etymology: Not stated, but Marenzeller (1885: 217) described the character of the opercular verticil spines for *Eupomatus exaltatus* as being elevated on a central column (“einer centralen Säule”). The Latin *exaltatus* ‘(up) lifted’ species-group name is perhaps in reference to this.

Evaluation: Gender-variable adjective, corrected to feminine ‘*exaltata*’ as recombined (e.g., Imajima 1976b: 232). Usages in *Hydroides* as ‘*exaltatus*’ exist (e.g., Dew 1959: 27).

Type locality: East coast of Enoshima Island (“Ostküste der Insel Eno-sima”), Sagami Bay, Honshu, Japan. There is an Enoshima-rettō Island, also off Honshu, but the Sagami Bay Enoshima Island (only ~0.5 km long) is the most likely visited.

Geolocation: 35.3008°, 139.4839° (map estimate).

WoRMS: 873938

Synonyms: No subjective synonyms.

***Hydroides exaltata vesiculosa* Fauvel, 1919 (originally as *Hydroides exaltatus* var. *vesiculosus***)

Status: Name now disused and representing a *species inquirenda*. Similarities of the original description to *Hydroides albiceps* have been noted, but the name is yet to be synonymised.

Etymology: Not stated, but *Hydroides exaltata vesiculosa* was evidently named for its vesicular dorsal verticil spine.

Evaluation: Gender-variable adjective with incorrect original ending, corrected to feminine (e.g., Monro 1937: 316).

Type locality: Gatavaké (Baie de Gatavaké), Mangareva Island, Mangareva/Gambier Islands, French Polynesia, South Pacific.

Geolocation: -23.1188°, -134.9798° (map estimate).

WoRMS: 875068

Synonyms: As *species inquirenda* has no synonyms.

***Hydroides externispina* Straughan, 1967b (original binomen)**

Etymology: Not stated, but for *Hydroides externispina* it is likely the Latin *spina* ‘thorn’ refers to the external (curved outwards) spines of the verticil.

Evaluation: Invariant noun in apposition.

Type locality: Heron Island, Queensland, Australia, collected close to the marine station by Dew (map in Straughan 1967b).

Geolocation: -23.4430°, 151.9110° (map estimate).

WoRMS: 328446

Synonyms: No subjective synonyms.

***Hydroides ezoensis* Okuda, 1934 (original binomen)**

Etymology: Not stated, but *Hydroides ezoensis* is evidently named after its area of collection, as Ezo (also as Yezo) is a former name for the island of Hokkaido, Japan. The species-group name ‘*ezoensis*’ has often been used for Japanese taxa, along with ‘*yezoensis*’.

Evaluation: Masculine/feminine invariant ‘-*ensis*’ adjective created from non-Latin geographic name.

Type locality: Not fixed by author. Original records are from “Akkeshi, Muroran, and Oshoro”, which are widely separated places around the coast of Hokkaido Island, with the first two having marine stations.

Geolocation: Imprecisely known (possibly as 43.0209°, 144.8368° for Akkeshi Marine Station).

WoRMS: 131003

Synonyms: See comments for *Hydroides diplochone*.

***Hydroides floridana* (Bush, 1910) (originally *Eupomatus floridanus*, new name for *Eupomatus uncinatus* non Philippi *sensu* Ehlers, 1887)**

Etymology: Not stated, but *Eupomatus floridanus* is evidently named after its purported region of collection. ‘Florida’ is Spanish for flowery land and is here combined with the Latin adjectival suffix -*anus* -*a* -*um*, indicating from Florida.

Evaluation: Gender-variable adjective based on a non-Latin geographic name, corrected to feminine herein. Usages in *Hydroides* as ‘*floridanus*’ exist (e.g., Bastida-Zavala and ten Hove 2002: 118) but not previously as ‘*floridana*’.

Type locality: Unknown, not certain to be off namesake Florida. When Ehlers (1887: 286) described the Polychaeta collected from voyages of Coast Survey Steamer “Blake” he wrote in his native German but recorded the two locations for the *Eupomatus* specimens literally in English as “inside fishing ground Cape Rear” and also “off W. down Cape Dear Rio” (both at 7 fathoms). However, these place-names seem to be misreadings as they could not be found in the Caribbean or Florida, nor do the “Blake” voyage reports include the names. The similarity of names suggests the location is possibly off Cape Fear, North Carolina, with its associated Cape Fear (Rio) River, disregarding that Ehlers’ monograph title appears to exclude Atlantic coast voyages the “Blake” also made. As the types are believed lost the original label cannot be checked. No specimens are currently listed in the Yale Peabody Museum online catalogue although Bush (1910: 498) earlier saw a mass of several hundred tubes, indicating an aggregation.

Geolocation: Unknown (if off Cape Fear, North Carolina then that place-name is at (gazetteer) 33.84°, -77.96°).

WoRMS: 369234

Synonyms: *Eupomatus decorus* Treadwell, 1931 (Grand Isle, Louisiana, Gulf of Mexico)

*Hydroides rostrata* Iroso, 1921 [junior objective synonym (same specimen)]

***Hydroides furcifera* (Grube, 1878b) (originally as *Serpula furcifera*)**

Etymology: Not stated but Grube described for *Serpula furcifera* forked spines in the opercular funnel as well as the verticil, thus Latin *furca* ‘fork’, combined with adjectival suffix -*fer* -*a* -*um* ‘bear’. Lewis and Short (1891: 795) include *furcifera* as a feminine noun meaning phallus, but it is unlikely this was Grube’s intention. A more common adjectival form would be *furcillata* ‘forked’.

Evaluation: Gender-variable adjective with correct original feminine ending. Names ending in -*fer* may be nouns or masculine adjectives (ICZN 1999, Article 31.2.2 example). The usage of Grube was adjectival as he used feminine -*fera*. A listing-only usage in *Hydroides* as ‘*furcifer*’ exists (ten Hove and Kupriyanova 2009: 53).

Type locality: “Ubay, Pandanon”, Philippines. Ubay (10.0606°, 124.4707°) is a small port on Bohol Island, and Pandanon Island (10.1779°, 124.0839°) is a small reef ~45 km to the west of Ubay.

Geolocation: 10.0606°, 124.4707° (map estimate, Ubay).

WoRMS: 369235

Synonyms: *Hydroides bifidus* [sic] Imajima, 1982 (off Arumonogui, Palau Islands, Micronesia)

***Hydroides fusca* Imajima, 1976a (original binomen)**

Etymology: Not stated, but the species-group name for *Hydroides fusca* from Latin *fuscus* ‘dark’ is evidently in reference to the “glossy black” verticil spines.

Evaluation: Gender-variable adjective with correct original feminine ending. A listing-only usage in *Hydroides* as ‘*fuscus*’ exists (ten Hove and Kupriyanova 2009: 53).

Type locality: Offshore east off northern tip of Tanegashima (island), Southern Japan, 80 m.

Geolocation: 30.8225°, 131.1335° (map estimate from author’s map).

WoRMS: 369236

Synonyms: No subjective synonyms.

***Hydroides fusicola* Mörch, 1863 (as Hydroides (Eupomatus) fusicola)**

Etymology: Not stated, but *Hydroides fusicola* is evidently named after the gastropod genus *Fusus* (now *Fusinus*) combined with -*cola* ‘dweller’, as it was found attached to a ‘*Fuso*’ sp.

Evaluation: Invariant compound noun in apposition with -*cola* as a substantival suffix. The Code has a stipulation (ICZN 1999, Article 30.1.4.2) that genera with -*cola* endings be treated as masculine compound nouns (or mostly so treated, similar to the -*oides* situation). It has no advice for species-group names with -*cola* suffixes, but they are recommended to be treated similarly (David and Gosselin 2002: 34), not declined to agree with the first noun or the genus.

Type locality: Japan (not further specified). Mörch only knew the specimen was from the collection of Wessel in Hamburg.

Geolocation: Unknown (gazetteer Japan central point as 37°, 138°).

WoRMS: 369237

Synonyms: *Hydroides okudai* Pillai, 1972 [*nom. n.* for “*Hydroides uncinata* (*sensu* Okuda et Uschakov)”] (location not fixed by author, but the Okuda (1937: 63) usage was for Ishihama, Japan, a name for at least four possible Honshu coastal locations)

***Hydroides gairacensis* Augener, 1934 (originally as Hydroides (Eupomatus) gairacensis)**

Status: Candidate *nomen protectum* against senior name Hydroides (Eupomatus) dunkeri Mörch, 1863 (*fide* Bastida-Zavala and ten Hove, 2002: 132). Prevailing usage maintained pending proof of sufficient usage of *Hydroides gairacensis*, but *Hydroides dunkeri* is a *nomen oblitum*, not used as a valid name after 1899 (listings are excluded as usages under ICZN 1999, Article 23.9.6).

Etymology: Not stated, but *Hydroides gairacensis* is evidently named after its place of collection, Gairaca.

Evaluation: Masculine/feminine invariant ‘-*ensis*’ adjective created from a non-Latin place-name.

Type locality: Gairaca, near Santa Marta, Caribbean Sea coast of Colombia.

Geolocation: 11.3184°, -74.1084° (map estimate).

WoRMS: 369238

Synonyms: Hydroides (Eupomatus) dunkeri Mörch, 1863 [*nomen oblitum*] (La Guayra, Panama, Caribbean Sea)

***Hydroides glandifera* Rioja, 1941a (originally as *Hydroides glandiferum*)**

Status: Type taxon by monotypy of *Olgaharmania* Rioja 1941b, a synonym of *Hydroides*.

Etymology: Not stated, but as Rioja (1941a: 174) writes of “una robusta protuberancia . . .en forma de glande” for *Hydroides glandifera*, it is likely to be a functional name for the bulbous dorsal verticil spine from Latin *glans* ‘acorn’ combined with adjectival suffix -*fer* -*a* -*um* ‘bear’. It is unclear why Rioja (incorrectly) used the neuter form ‘*glandiferum*’ at first, but he later (Rioja 1941b: 733) modified the spelling for his feminine *Olgaharmania glandifera* combination.

Evaluation: Gender-variable adjective with corrected feminine ending. Usages in *Hydroides* as ‘*glandifer*’ and ‘*glandiferum*’ exist (e.g., Bastida-Zavala and ten Hove 2003: 89).

Type locality: Caleta (Playa Caleta), Acapulco, Mexico.

Geolocation: 16.8313°, -99.9031° (map estimate).

WoRMS: 338016

Synonyms: No subjective synonyms.

***Hydroides glasbyi* Sun, Wong, ten Hove, Hutchings, Williamson & Kupriyanova, 2015 (original binomen)**

Etymology: The authors dedicated *Hydroides glasbyi* to Christopher J. Glasby.

Evaluation: Invariant genitive form *glasbyi* of the personal name Glasby.

Type locality: Fort Hill Wharf, Darwin, Northern Territory, Australia.

Geolocation: -12.4714°, 130.8467° (authors, 12°28'17"S, 130°50'48"E).

WoRMS: 852813

Synonyms: No subjective synonyms.

***Hydroides gracilis* (Bush, 1905) (originally as *Eupomatus gracilis*)**

Etymology: Not stated, but the Latin *gracilis* ‘slender’ name for *Hydroides gracilis* is likely referring to the simple verticil spines.

Evaluation: Invariant adjective (masculine/feminine ‘*gracilis*’).

Type locality: Pacific Grove, California, Pacific coast USA.

Geolocation: 36.6236°, -121.9119° (map estimate).

WoRMS: 333640

Synonyms: *Eupomatus intereans* Chamberlin, 1919 (Laguna Beach, California coast)

***Hydroides gradata* Straughan, 1967a (original binomen)**

Status: The synonymy of *Hydroides basispinosa* and *Hydroides gradata* Straughan, 1967a with *Hydroides operculata* Treadwell, 1929 was followed by Sun et al. (2015: 63), but is being re-examined, and we provisionally include the *Hydroides gradata* record separately.

Etymology: Not stated, but for *Hydroides gradata* the Latin *gradata* ‘gradual’ is evidently describing the gradual size change of the ring of opercular spines.

Evaluation: Gender-variable adjective with correct original feminine ending.

Type locality: Pretty Beach, 40 km north of Cairns, Queensland, Australia

Geolocation: -16.6111°, 145.5318° (map estimate).

WoRMS: 384604

Synonyms: See *Hydroides operculata* comments.

***Hydroides helmata* (Iroso, 1921) (originally as *Eupomatus helmatus*)**

Status: Zibrowius (1971: 713–714) synonymised an older name, *Eupomatus affinis* Marion, 1875, under *Hydroides helmata*. This is not possible on priority, nor does *Hydroides affinis* qualify as a *nomen oblitum* as it was used as valid (Zibrowius 1968: 115) post 1899. We include both names (see entry for *Hydroides affinis* as *species inquirenda*).

Etymology: Not stated, but *Eupomatus helmatus* is likely named after the larger helmet-like dorsal verticil spine as Iroso (1921: 54) describes “che ricade sugli altri ad elmo” (which falls on others [spines] helmet-like). Helm and helmet are not from Latin, though the author’s construction appears to be intended as adjectival, with adjectival suffix -*atus* added to mean helm-like.

Evaluation: Gender-variable adjective corrected to feminine in *Hydroides* in Zibrowius (1971: 713).

Type locality: Unspecified Gulf of Naples (Golfo di Napoli), Italy.

Geolocation: Imprecisely known (a Golfo di Napoli mid-point (gazetteer) is 40.8°, 14.2°).

WoRMS: 131004

Synonyms: No subjective synonyms, but has been linked to *Hydroides affinis* (see above).

***Hydroides heterocera* (Grube, 1868) (originally as Serpula (Eupomatus) heterocerus)**

Etymology: Not stated but the name for *Serpula heterocerus* is likely describing the dimorphism in verticil spines, from Greek hετεροσ (*heteros*) ‘different’ and κερασ (*keras*) ‘horn’. The Latinized *heterocerus* is an adjectival form to be declined.

Evaluation: Gender-variable adjective corrected to feminine in Zibrowius (1971: 715). Grube (1868: 639) originally incorrectly created a masculine ‘*heterocerus*’ in agreeing with the masculine subgenus *Eupomatus* rather than the feminine genus *Serpula*. Usages exist in *Hydroides* as ‘*heterocerus*’ (e.g., Ben Eliahu and ten Hove 2011: 26), and as the misspelling ‘*heteroceros*’ (e.g., Day 1967: 807).

Type locality: Unspecified Red Sea. Grube’s report title refers to Red Sea worms collected by Georg Ritter von Frauenfeld. Grube states in his opening sentence that the worms were handed to him without any other information, and it seems he did not investigate this further. In the narrative of his visit von Frauenfeld (1855) mentions Suez, the Sinai Peninsula, and seeing countless annelids on the Red Sea shore, but he does not match observation to locality.

Geolocation: Imprecisely known, but perhaps northern Red Sea (a gazetteer Red Sea mid-point is 20.3°, 38.6°).

WoRMS: 851900

Synonyms: No subjective synonyms. However, the misidentification Serpula (Hydroides) uncinata non Philippi, *sensu* Gravier, 1906, has been assigned to *Hydroides heterocera* (e.g., Pixell 1913: 75).

***Hydroides heterofurcata* Pillai, 1971 (original binomen)**

Etymology: Not stated, but evidently *Hydroides heterofurcata* is named because there are two types of furcate verticil spines of the operculum (Pillai 1971: 114).

Evaluation: Gender-variable adjective with correct original feminine ending. Usages as ‘*heterofurcatus*’ exist (e.g., ten Hove and Kupriyanova 2009: 53).

Type locality: near Talaimannar Pier, Sri Lanka, 4 m depth.

Geolocation: 9.1079°, 79.7292° (map estimate from author map).

WoRMS: 328449

Synonyms: No subjective synonyms.

***Hydroides hexagona* (Bosc, 1802) (originally *Serpula hexagona*)**

Status: A name disused by taxonomists and representing a *species inquirenda*. The original description and figure are rudimentary and the species Bosc saw will remain indeterminable unless original specimens are found (unlikely). However, the name cannot be a *nomen oblitum* as it was revived as *Hydroides hexagonus* [sic] in three widely used manuals on invertebrates of the United States eastern coast (Pratt 1916, Grave 1937, Costello et al. 1957). These instances should be considered misidentifications, and might be referable either to the junior name *Hydroides dianthus* (*fide* Zibrowius 1971: 697, Bastida-Zavala and ten Hove 2002: 108), or to other similar species. Nevertheless, there are multiple modern citations of the research on *Hydroides* sperm (e.g., Colwin and Colwin 1961) in which the name appeared.

Etymology: Bosc described the tube of *Serpula hexagona* as “montrant la moitié d’un prisme hexagone …”, and the name is a New Latin adjectival form for six-sided, modified from Greek. Bosc’s figure shows two ridges so the tube cross-section would be trapezoidal, not literally hexagonal as named, but half (la moitié) of that.

Evaluation: Gender-variable adjective with correct feminine ending herein. Usages in *Hydroides* as ‘*hexagonus*’ and ‘*hexagonis*’ exist (e.g., Pratt 1916: 302, Grave and Oliphant 1930: 234) but not previously as ‘*hexagona*’.

Type locality: Charleston Harbour, Charleston, South Carolina, Atlantic coast USA.

Geolocation: 32.8186°, -79.9279° (gazetteer).

WoRMS: 384606

Synonyms: As *species inquirenda* has no synonyms.

***Hydroides homoceros* Pixell, 1913 (original binomen)**

Etymology: Not stated, but for *Hydroides homoceros* it is likely that the Greek hομοσ (*homos*) ‘uniform’ and κερασ (*keras*) ‘horn’, refers to the opercular verticil spines. Pixell appears to have named ‘*homoceros*’ as the opposite to ‘*heteroceros*’ (her error for the existing ‘*heterocerus*’) which she mentions.

Evaluation: Incorrect Latinization treated here as an unchanging noun in apposition. Usages exist as ‘*homocera*’ (e.g., Ben-Eliahu and ten Hove 1992: 35) and ‘*homocerus*’ (e.g., Bellan 2001: 226).

Type locality: Multiple Indian Ocean localities as the syntypes (aggregated as only one NHM specimen lot 1924.6.13.147 received from the Cyril Crossland Collection) came both from the Maldive area (specified as Miladhunmadulu Atoll and Minikoi), and from off Zanzibar.

Geolocation: Unknown (map estimate 6.02°, 73.19° for Noonu, the southern Miladhunmadulu Atoll).

WoRMS: 238212

Synonyms: No subjective synonyms.

***Hydroides huanghaiensis* Sun & Yang, 2000 (original binomen)**

Etymology: Not stated, but *Hydroides huanghaiensis* is evidently named after the sea in which the worm was collected as “Huanghai” means Yellow Sea in Chinese.

Evaluation: Masculine/feminine invariant ‘-*ensis*’ adjective created from a non-Latin geographic name, Huanghai.

Type locality: Northern Yellow Sea, off the Chinese coast near Dalian.

Geolocation: 39.00°, 122.1167° (as authors, 39°00'N, 122°70'E [? error for 7']).

WoRMS: 328450

Synonyms: No subjective synonyms.

***Hydroides humilis* (Bush, 1905) (originally as *Eupomatus humilis*)**

Etymology: Not stated, but for *Eupomatus humilis* possibly the name, from Latin *humilis* ‘humble’ (or ‘low’), is referring to the small size of the single specimen collected.

Evaluation: Masculine/feminine invariant adjective (*humilis* -*e*) (Stearn 1983: 94).

Type locality: Guaymas, Gulf of California coast, Sonora state, Mexico. Bush provides no other details other than the name Guaymas (Mexico).

Geolocation: 27.9087°, -110.8931° (map estimate).

WoRMS: 369239

Synonyms: No subjective synonyms.

***Hydroides inermis* Monro, 1933 (original binomen)**

Etymology: Not stated, but for *Hydroides inermis* it is likely that the Latin *inermis* ‘unarmed’, is referring to the verticil spines without spinules. Monro stated the operculum “lacks spines both on the lower and the upper calix”.

Evaluation: Masculine/feminine invariant adjective (*inermis* -*e*).

Type locality: James Bay, Isla Santiago (was James Island), Galapagos, Ecuador.

Geolocation: -0.1959°, -90.8424° (map estimate).

WoRMS: 338017

Synonyms: No subjective synonyms.

***Hydroides inornata* Pillai, 1960 (original binomen)**

Status: The current synonymy of *Hydroides inornata* with *Hydroides operculata* is being re-evaluated, and meantime it is included separately here.

Etymology: Not stated, but for *Hydroides inornata* it is likely that the Latin *inornatus* ‘unadorned’ is referring to the verticil spines without side spinules.

Evaluation: Gender-variable adjective with correct original feminine ending. Usages as ‘*inornatus*’ exist (e.g., Amoureux et al.: 57).

Type locality: Maha Alamba (not found, perhaps disused), “about a mile” from the Negombo Lagoon entrance (an aquatic research institute is nearby), north of Colombo, west coast of Sri Lanka.

Geolocation: 7.1945°, 79.8392° (map estimate).

WoRMS: 338018

Synonyms: No subjective synonyms, and has been regarded as junior to *Hydroides operculata* (e.g., Sun et al. 2015: 62).

***Hydroides kimberleyensis* Pillai, 2009 (original binomen)**

Etymology: The author named *Hydroides kimberleyensis* after the Kimberley region of Western Australia.

Evaluation: Masculine/feminine invariant ‘-*ensis*’ adjective created from a non-Latin geographic name.

Type locality: Off east side of Fenelon Island (main island of Institut Islands) at 6 m, Kimberley, Western Australia.

Geolocation: -14.1167°, 125.7167° (author).

WoRMS: 555195

Synonyms: No subjective synonyms.

***Hydroides lambecki* Bastida-Zavala & ten Hove, 2002 (original binomen)**

Etymology: The authors named *Hydroides lambecki* after Hugh J.P. Lambeck (entomologist, deceased, one time assistant to ten Hove), who first noted this as a species different from *Hydroides mongeslopezi*.

Evaluation: Invariant genitive form *lambecki* of the personal name Lambeck.

Type locality: Vaarsenbaai (cove), Boca Sami, Curaçao, Netherlands Antilles.

Geolocation: 12.15°, -69.00° (gazetteer).

WoRMS: 328452

Synonyms: No subjective synonyms.

***Hydroides lirs* Kupriyanova, Sun, ten Hove, Wong & Rouse, 2015 (original binomen)**

Etymology: The authors named *Hydroides lirs* after the Australian Museum’s Lizard Island Research Station (LIRS).

Evaluation: Invariant non-Latinized noun in apposition ‘*lirs*’ from an acronym, pronounced as a single word.

Type locality: Front of reef between Bird and South Islands, Lizard Island, Queensland, Australia, -14.6978°, 145.4639° (station MI QLD 2354 in Ribas and Hutchings, 2015).

Geolocation: -14.6978°, 145.4639° (station list).

WoRMS: 877990

Synonyms: No subjective synonyms.

***Hydroides longispinosa* Imajima, 1976b (original binomen)**

Etymology: Not stated, but *Hydroides longispinosa* evidently is named after the “conspicuous, long central spine” (long in comparison with *Hydroides elegans*), based on Latin adjectives *longus* ‘long’ with *spinosus* ‘spined’.

Evaluation: Gender-variable adjective with correct original feminine ending. Usages as ‘*longispinosus*’ exist (e.g., Bailey-Brock 1987: 282).

Type locality: Koniya, Amami-Oshima, Amami Islands, Southern Japan.

Geolocation: 28.1472°, 129.3078° (map estimate).

WoRMS: 328453

Synonyms: *Hydroides centrospina* Wu & Chen, 1981 (Yulin Harbour, Hainan Island, South China Sea)

***Hydroides longistylaris* Chen & Wu, 1980 (original binomen)**

Etymology: Not stated, but for *Hydroides longistylaris* evidently the Latin *longus* ‘long’ and adjectival Latinization of Greek στυλος (stylos) ‘pillar’ refers to the long, elongated basis of the opercular funnel, thus ‘pillar-like’.

Evaluation: Masculine/feminine invariant adjective (-*stylaris* -*e*).

Type locality: Shellfish farms, Zhangpu (Zhangzhou), Fujian Province, China.

Geolocation: 24.4379°, 117.9762° (map estimate).

WoRMS: 328454

Synonyms: No subjective synonyms.

***Hydroides malleolaspina* Straughan, 1967a (original binomen)**

Etymology: Not stated, but the name for *Hydroides malleolaspina* is evidently a compound noun from Latin *malleolus* ‘small hammer’, referring to the dorsal hammer-shaped verticil spine, and *spina* ‘thorn’.

Evaluation: Invariant noun in apposition. Usages as ‘*malleolaspinus*’ exist (e.g., Murray et al. 2010: 393).

Type locality: Pialba, Hervey Bay, Queensland, Australia.

Geolocation: -25.2747°, 152.8345° (map estimate).

WoRMS: 369240

Synonyms: “*Hydroides trihamulatus*” [sic] Pillai, 2009 [unavailable name (no type-designation), assignment by Murray et al. 2010] (Australia)

***Hydroides microtis* Mörch, 1863 (originally as Hydroides (Eucarphus) microtis)**

Etymology: Not stated, but for *Hydroides microtis* the ‘*micro*’ derives from Greek μικρος (micros) ‘small’, and perhaps is combined with Greek neuter noun genitive οτοσ (otos) ‘ear’. The Latinizations ‘*microtis*’ and ‘*microtus*’ are in use as both genus and species-group names for small-eared biota. Whether the same derivation applies for *Hydroides microtis* is unclear, as the verticil spines are knob-tipped and not notably small or ear-like.

Evaluation: Invariant whether a noun in apposition or (masculine/feminine) intended as adjectival.

Type locality: North America (unspecified) as “*ad Americam borealem*” on *Argopecten irradians* (was as *Pecten*), collected by A. B. Mayer, presumably on the Atlantic coast as *Argopecten irradians* is the bay scallop of that region.

Geolocation: Unknown (unspecified Atlantic coast of North America, with 44°, -68° the mid point of the coastal extent).

WoRMS: 333641

Synonyms: No subjective synonyms.

***Hydroides minax* (Grube, 1878b) (originally as *Serpula minax*)**

Etymology: Not stated, but for *Serpula minax* the Latin adjective *minax* -*acis* meaning ‘jutting out’ is likely referring to the enormous dorsal verticil spine.

Evaluation: Invariant adjective (masculine/feminine ‘*minax*’).

Type locality: Philippines (unspecified).

Geolocation: Unknown (12°, 122° (gazetteer) is central to the Philippines Islands).

WoRMS: 131007

Synonyms: Serpula (Hydroides) monoceros Gravier, 1906 (Bonhoure Recif, Djibouti, Gulf of Aden)

***Hydroides mongeslopezi* Rioja, 1958 (original binomen)**

Etymology: The author named *Hydroides mongeslopezi* after Ricardo Monges López of Veracruz.

Evaluation: Invariant genitive noun *mongeslopezi* from modern personal name of Monges López.

Type locality: On floating pumice, Playa Norte, Isla Santiaguillo, Veracruz, Gulf of Mexico.

Geolocation: 19.1634°, -95.8502° (map estimate).

WoRMS: 328456

Synonyms: No subjective synonyms.

***Hydroides monroi* Zibrowius, 1973 (original binomen)**

Etymology: Not stated, but the species *Hydroides monroi* is evidently named after C. C. A. (Charles Carmichael Arthur) Monro, who had studied the specimens earlier.

Evaluation: Invariant genitive noun *monroi* from modern personal name of Monro.

Type locality: Pointe Noire, Congo, West Africa.

Geolocation: -4.7858°, 11.8361° (map estimate).

WoRMS: 328457

Synonyms: No subjective synonyms.

***Hydroides mucronata* Rioja, 1958 (original binomen)**

Etymology: Not stated, but the name for *Hydroides mucronata* is evidently referring to the pointed (Latin *mucronatus*) side spines of the verticil spines “que tienen forma de mucron” (Rioja 1958: 256).

Evaluation: Correct original adjectival feminine ending. Usages as ‘*mucronatus*’ exist (e.g., Bastida-Zavala and ten Hove 2002: 141).

Type locality: Isla de Sacrificios, Veracruz, Gulf of Mexico.

Geolocation: 19.1749°, -96.0929° (map estimate).

WoRMS: 328458

Synonyms: No subjective synonyms.

***Hydroides multispinosa* Marenzeller, 1885 (original binomen)**

Etymology: Not stated, but the name for *Hydroides multispinosa* evidently refers adjectivally to multiple lateral spinules on the verticil spines.

Evaluation: Correct original adjectival feminine ending. Usages as masculine ‘*multispinosus*’ exist (e.g., ten Hove and Kupriyanova 2009: 54).

Type locality: Shore at Eno-sima (Enoshima), Sagami Bay, Honshu, Japan. There is an Enoshima-rettō Island, also off Honshu, but the Sagami Bay Enoshima is the most likely visited.

Geolocation: 35.2977°, 139.4817° (map estimate).

WoRMS: 335316

Synonyms: No subjective synonyms.

***Hydroides nanhaiensis* Wu & Chen, 1981 (original binomen)**

Etymology: Not stated, but *Hydroides nanhaiensis* is evidently named broadly geographically as “Nanhai” is the South China Sea in Chinese.

Evaluation: Masculine/feminine invariant ‘-*ensis*’ adjective created from a non-Latin geographic area name.

Type locality: Xi River estuary, Pearl River Delta, Macao, Guangdong, China coast, South China Sea, 58m, fixed on rock, stations 6016, 6044 (*fide* Sun and Yang 2014: 218 (map), 241; no locality in the original text).

Geolocation: 22.0602°, 113.4792° (map estimate, Xi River mouth).

WoRMS: 328459

Synonyms: No subjective synonyms.

***Hydroides nigra* Zibrowius, 1971 (original binomen)**

Etymology: Not stated, but the name for *Hydroides nigra* is evidently referring to the dark colour of the operculum, especially of the opercular constriction (“un anneau noir à la base de l‘opercule”) and the verticil spines, and derived from the Latin adjective *niger*, *nigra*, *nigrum* ‘black’.

Evaluation: Gender-variable adjective with correct original feminine ending. Usages as ‘*niger*’ exist (e.g., Bellan 2001: 226)).

Type locality: Tabarka “au large de l’ile [Tabarka] et de la Pointe Meloula [4 km west]”, Tunisia, Mediterranean Sea.

Geolocation: 36.9666°, 8.7588° (map estimate for north end of Tabarka).

WoRMS: 328460

Synonyms: No subjective synonyms.

***Hydroides nikae* Sun, Wong, Tovar-Hernández, Williamson & Kupriyanova, 2016 (original binomen)**

Etymology: The authors named *Hydroides nikae* after Nika Mikhin, daughter of Kupriyanova.

Evaluation: Invariant feminine genitive form *nikae* of given name Nika.

Type locality: Edithburgh Jetty, Edithburgh, St Vincent Gulf, South Australia.

Geolocation: -35.0848°, 137.7488° (adjusted to jetty from authors’ inland 35°05'S, 137°44' (should be 45') E).

WoRMS: 871949

Synonyms: No subjective synonyms.

***Hydroides nodosa* Straughan, 1967a (original binomen)**

Etymology: Not stated, but *Hydroides nodosa* is likely named for the internal “rounded projection” at the base of each verticil spine, from the adjective *nodosus* -*a* -*um* ‘knotty’.

Evaluation: Gender-variable adjective with correct original feminine ending. Usages as ‘*nodosus*’ exist (e.g., ten Hove and Kupriyanova 2009: 54).

Type locality: Tannum Sands, Gladstone, Queensland, Australia.

Geolocation: -23.93°, 151.37° (map estimate *fide* Australian Museum holotype W.4013 catalogue record).

WoRMS: 328461

Synonyms: No subjective synonyms.

***Hydroides norvegica* Gunnerus, 1768 (original binomen)**

Status: The type species of the genus (by monotypy).

Etymology: Not stated, but the name for *Hydroides norvegica* is evidently derived from the country of collection, Norway (Latin *Norvegia*), from which the feminine-suffix adjective ‘*norvegica*’ is derived.

Evaluation: Gender-variable adjective based on a geographic name. *Hydroides norvegica* was given a species-group name with a feminine ending. Many usages as ‘*norvegicus*’ exist (e.g., Moen 2006: 115).

Type locality: Trøndelag region, Norway. Trondheimsfjord off Statsbygd is one of three locations mentioned by Gunnerus (see Moen, 2006: 118).

Geolocation: Imprecisely known (map estimate 63.4687°, 10.011° for off Statsbygd).

WoRMS: 131009

Synonyms: There is an extensive list by McIntosh (1923: 347) of early serpulid names and usages in *Eupomatus*, *Hydroides*, *Serpula*, and *Vermilia* that are suggested to be *Hydroides norvegica* synonyms. Nine of the placements were repeated later in a world catalogue (Hartman 1959), but only two can be confirmed here (see Read and Fauchald 2016 for status of the remainder). Also Mörch, 1863 named a subspecies *Hydroides norvegica gronlandica*, based on a Fabricius MS, but it is a *nomen dubium* unlikely to be a *Hydroides*.

*Eupomatus trypanon* Claparède, 1870b (Gulf of Naples, Italy, Tyrrhenian Sea)

*Serpula solitaria* Bean, 1844 (Scarborough, North Yorkshire, England)

***Hydroides novaepommeraniae* Augener, 1925 (originally as Hydroides (Eupomatus) novae-pommeraniae)**

Etymology: Not stated, but the name for *Hydroides novaepommeraniae* is evidently a Latinized form of the former name of the island of collection, New Britain, Bismarck Archipelago, now part of Papua New Guinea, once a German colony named Neupommern, after the Baltic (Ostsee) coastal lands besides Pommersche Bucht.

Evaluation: Invariant noun in the genitive case created from a non-Latin geographic name Latinized as ‘*novaepommeran*’.

Type locality: “Hanam-Hafen” (Hannan or Garua Harbour), north coast of New Britain, Papua New Guinea.

Geolocation: -5.2833°, 150.0333° (map estimate).

WoRMS: 131010

Synonyms: *Hydroides grubei* Pillai, 1965 (Binakayan, Cavite, Manila Bay, Philippines)

***Hydroides ochotereana* Rioja, 1941a**

Etymology: Rioja (1941a: 167) stated the name for *Hydroides ochotereana* was “dedicar esta especie al Maestro D. Isaac Ochoterena”, but he used the spelling ‘*ochotereana*’ for the species-group name.

Evaluation: Incorrect Latinization to be treated as a noun in apposition. The use of *Hydroides ochotereana* has been regarded as an accidental incorrect original spelling by Bastida-Zavala and ten Hove (2003), who cited Article 32.5 (ICZN 1999) as justification for using ‘*ochoterena*’, although that would be an unchanged noun in apposition, rather than a genitive. Instead, we cannot reject the likelihood that Rioja had intentionally used the altered ‘-*eana*’ ending (after all he used it consistently five times but correctly spelled the name of dedicatee Ochotorena) aiming to create an adjectival form of Ochotorena. His adaptation could be intended as a rendering using the suffix ‘-*anus*’ -*ana*’ (belonging to), frequently used for adjectival Latinization of nouns based on personal and geographic names. As it was the author who was responsible for an incorrect Latinization (ICZN 1999, Article 32.5.1) his original spelling is not corrected (also see Welter-Schultes 2013: 77). This also avoids the name looking like an authorship (ICZN 1999, recommendation 31A).

Type locality: La Aguada and La Quebrada beaches, Acapulco, Mexico.

Geolocation: 16.8461°, -99.9156° (La Quebrada, map estimate).

WoRMS: 328462

Synonyms: No subjective synonyms.

***Hydroides operculata* (Treadwell, 1929) (originally as *Eupomatus operculata* [sic])**

Etymology: Not stated, but the name for *Eupomatus operculata* derives from the Latin verb *operculo* -*avi* -*atum* ‘to cover’, and in New Latin *operculata* is used as an adjectival form. It is unclear why Treadwell chose the name as all *Hydroides* have opercula. His specimen was endowed with two, but he didn’t name it ‘*bioperculata*’.

Evaluation: Gender-variable adjective with incorrect original feminine ending for *Eupomatus*. Usages in *Hydroides* as ‘*operculatus*’ exist (e.g., Bellan 2001: 226).

Type locality: Berbera, Somaliland, Gulf of Aden.

Geolocation: 10.441°, 45.0075° (map estimate).

WoRMS: 131011

Synonyms: *Hydroides basispinosa* Straughan, 1967a [re-evaluating, see listing herein]

*Hydroides gradata* Straughan, 1967a [re-evaluating, see listing herein]

*Hydroides inornata* Pillai, 1960 [re-evaluating, see listing herein]

***Hydroides panamensis* Bastida-Zavala & ten Hove, 2003 (original binomen)**

Etymology: The authors state that *Hydroides panamensis* is named “for its distribution, as far as known yet restricted to the Pacific side of Panama (and adjacent areas).”

Evaluation: Masculine/feminine invariant ‘-*ensis*’ adjective created from a non-Latin geographic name.

Type locality: Paitilla Beach (Punta Paitilla), Panama City, Western Panama.

Geolocation: 8.9733°, -79.5183° (map estimate).

WoRMS: 328464

Synonyms: No subjective synonyms.

***Hydroides parva* (Treadwell, 1902) (originally as *Eupomatus parvus*)**

Etymology: Not stated, but Treadwell (1902: 210) stated the specimens of *Eupomatus parvus* were “very small” (6 mm) thus Latin *parvus* ‘small’.

Evaluation: Gender-variable adjective recombined in *Hydroides* with correct feminine ending (e.g., Zibrowius 1971: 712, 717). Usages in *Hydroides* as ‘*parvus*’ exist (e.g., Hartman 1956: 250).

Type locality: West coast of Puerto Rico, Caribbean Sea, at both Boqueron Bay and nearby Mayagüez Harbour (station 6062, estimated 18.2°, -67.17°), as Treadwell had specimens from both locations. No station geolocations appear to have been available for the various *Fish Hawk* ‘Porto Rico’ stations (Treadwell 1939). Syntypes (USNM 16173) in the Smithsonian National Museum of Natural History are recorded as from Boqueron Bay (Bahia de Boqueron).

Geolocation: 18.0208°, -67.1987° (map estimate, Bahia de Boqueron).

WoRMS: 876557

Synonyms: No subjective synonyms.

***Hydroides pectinata* (Philippi, 1844) (originally as *Eupomatus pectinatus*)**

Status: Name now disused and representing a *species inquirenda*. It is not eligible as a candidate *nomen oblitum* (used in taxonomy by Iroso 1921: 49, Naples), but is indeterminable unless original specimens are found. The operculum figured by Philippi is similar to that of *Hydroides elegans* (Haswell, 1883) (*fide* Zibrowius 1971: 718).

Etymology: Philippi’s brief Latin description of *Eupomatus pectinatus* describes the operculum spines as ‘*utrinque pectinatis*’ (pectinate both sides) with three sharp teeth. The Latin adjective *pectinatus* indicates comb-like divisions.

Evaluation: Gender-variable adjective recombined in *Hydroides* with correct feminine ending (e.g., Mörch 1863: 377).

Type locality: Unspecified Mediterranean, but can be narrowed to the Tyrrhenian Sea coast of Italy as Philippi’s activities were in western Italy, and plausibly to Naples as he was based there prior to 1844.

Geolocation: Unknown (Tyrrhenian Sea, with Naples shore (40.8327°, 14.2358° map estimate) a possible point location).

WoRMS: 393822

Synonyms: As *species inquirenda* has no synonyms although *Hydroides elegans* has been suggested.

***Hydroides perezi* Fauvel, 1918 (original binomen)**

Etymology: Fauvel announces on the first page of his article that *Hydroides perezi* is dedicated to “M. Ch. Pérez”, who collected the worms off the Arabian coast.

Evaluation: Invariant genitive form *perezi* from personal name Pérez.

Type locality: Pearling banks (within 24°55'N–25°10'N, 54°40'E–55°10'E) dredged ~15 miles from the coast of Oman (currently near Dubai, UAE) (Fauvel 1918: 329).

Geolocation: 25.0417°, 54.9167° (map estimate, mid point of bounds given by author).

WoRMS: 209947

Synonyms: No subjective synonyms.

***Hydroides plateni* (Kinberg, 1867) (originally as *Eupomatus plateni*)**

Etymology: Not stated, but *Eupomatus plateni* is evidently named after its La Plata collection station of the Swedish frigate *Eugenie* expedition.

Evaluation: Invariant genitive from Old Frankish ‘platen’, ultimately from Greek πλατuσ (platus) ‘flat’, relating to the Spanish La Plata placename, which plausibly had derived from a once widespread use of ‘plate’ to signify precious metals. Other ‘*plateni*’ species group names of the period may relate to the German zoological collector Carl Platen (1843–1899) but clearly not this one.

Type locality: Offshore off the La Plata (“*prope ostium fluvii* La Plata”) embayment, Argentina/Uruguay (the *Eugenie* berthed at Montevideo, Uruguay).

Geolocation: Imprecisely known (map estimate -35.3°, -56.3° for mid La Plata, offshore of Montevideo).

WoRMS: 369242

Synonyms: No subjective synonyms.

***Hydroides protulicola* Benedict, 1887 (original binomen)**

Etymology: Not stated, but *Hydroides protulicola* is evidently named from *Protula* (serpulid genus) combined with -*cola* ‘dweller’, because it was fastened on the tube of *Protula diomedeae* Benedict, 1887.

Evaluation: Invariant compound noun in apposition with -*cola* as a substantival suffix.

Type locality: Northeast off Cape Hatteras, North Carolina, Atlantic coast USA, 86 m.

Geolocation: 35.7°, -74.9083° (from author as 35°42'00"N, 74°54'30"W).

WoRMS: 338020

Synonyms: No subjective synonyms.

***Hydroides pseudexaltata* Pillai, 2009 (originally as *Hydroides pseudexaltatus*)**

Etymology: The author states he named *Hydroides pseudexaltatus* after the superficial similarity of the operculum to that of *Hydroides exaltatus*.

Evaluation: Gender variable adjective, with usage as corrected feminine ‘*pseudexaltata*’ in Sun et al. (2015: 65).

Type locality: Shoreline on “island off north east Heywood Island” Kimberley, Western Australia. The author’s given geolocation (15°05'S, 124°25'E) is oceanic and clearly incorrect. This is not a rounding error. The island north east of Heywood is the closely adjacent and much larger Jungulu Island.

Geolocation: -15.3167°, 124.3493° (map estimate, Jungulu shore adjacent Heywood Island).

WoRMS: 882697

Synonyms: No subjective synonyms.

***Hydroides pseudouncinata* Zibrowius, 1968 (original binomen)**

Status: Currently valid but it is possibly the same as the disused *Hydroides euplaeana* (see above).

Etymology: Not stated, but evidently *Hydroides pseudouncinata* was named because it represents one of the taxa previously confounded under *Hydroides uncinata* (see below), a name regarded as of indeterminable identity from its original description (*fide* Zibrowius 1971: 709).

Evaluation: Gender-variable adjective with correct original feminine ending. Usages as ‘*pseudouncinatus*’ species (or nominal subspecies) exist (e.g., ten Hove and Kupriyanova 2009: 54).

Type locality: East off Île Gaby (also Degaby), Marseille, France, Mediterranean Sea (not in Zibrowius 1968, *fide* Zibrowius 1971: 708).

Geolocation: 43.2776°, 5.3449° (map estimate).

WoRMS: 131012

Synonyms: No subjective synonyms.

***Hydroides pseudouncinata africana* Zibrowius, 1971 (original trinomen)**

Etymology: The author named subspecies *Hydroides pseudouncinata africana* after its continent of collection, Africa.

Evaluation: Gender-variable adjective with correct original feminine ending. Usages as ‘*africanus*’ exist (e.g., ten Hove and Kupriyanova 2009: 54).

Type locality: Off Rio de Oro, Mauritania, Atlantic coast of Africa.

Geolocation: 21.0833°, -17.4° (author, 21°05'N, 17°24'W).

WoRMS: 335489

Synonyms: No subjective synonyms.

***Hydroides qiui* Sun, Wong, ten Hove, Hutchings, Williamson & Kupriyanova, 2015 (original binomen)**

Etymology: The authors dedicated *Hydroides qiui* to Jian-Wen Qiu.

Evaluation: Invariant genitive form *qiui* from personal name Qiu.

Type locality: East Arm Port, Darwin Harbour, Northern Territory, Australia.

Geolocation: -12.4917°, 130.8831° (authors, 12°29'30"S, 130°52'59"E).

WoRMS: 852783

Synonyms: No subjective synonyms.

***Hydroides ralumiana* Augener, 1927 (originally (incorrectly) as Hydroides (Eupomatus) ralumianus)**

Etymology: Not stated, but *Hydroides ralumianus* is named after Ralum plantation, near its place of collection.

Evaluation: Gender-variable adjective, based on a non-Latin place-name, corrected by Day (1967: 806) from the masculine. The suffix ‘-*anus* -*a*’ is frequently used for Latinization of names based on localities and personal names.

Type locality: Ralum, Kokopo, Blanche Bay, New Britain (Neu-Pommern), Bismarck Archipelago of Papua New Guinea. The plantation “Ralum” was briefly the base for Friedrich Dahl, who collected the worms in 1896–97 (*fide* Augener 1927).

Geolocation: -4.3371°, 152.2674° (map estimate).

WoRMS: 209951

Synonyms: No subjective synonyms.

***Hydroides recta* Straughan, 1967a (original binomen)**

Etymology: Not stated, but the name for *Hydroides recta* is perhaps a reference to the 8^th^ enlarged dorsal verticil spine with its “pointed process perpendicular to it” from Latin *rectus* ‘perpendicular’.

Evaluation: Gender-variable adjective with correct original feminine ending. Usages as masculine ‘*rectus*’ exist (e.g., Pillai 2009: 132).

Type locality: Pretty Beach, north of Cairns, Queensland, Australia.

Geolocation: -16.6111°, 145.5318° (map estimate, a beach 40 km north of Cairns).

WoRMS: 328466

Synonyms: No subjective synonyms.

***Hydroides recurvispina* Rioja, 1941a (original binomen)**

Etymology: Not stated, but the name for *Hydroides recurvispina* is likely referring to the verticil spines which are sharply curving backwards on themselves. Thus the name is formed from Latin *recurvus* ‘backward curved’ combined with *spina* ‘thorn’.

Evaluation: Invariant noun in apposition. Bastida-Zavala and ten Hove (2003: 99) maintained the original spelling.

Type locality: La Aguada, Acapulco, Mexico.

Geolocation: 16.8398°, -99.9009° (map estimate).

WoRMS: 328467

Synonyms: No subjective synonyms.

***Hydroides rhombobula* Chen & Wu, 1980 (originally as *Hydroides rhombobulus*)**

Etymology: Not stated, but the name for *Hydroides rombobulus* may be referring to the shape of the verticil spines, derived from a combination of Greek ρομβος (rombos) ‘*rhombus*’, which is a parallelogram with only opposite angles equal, and Latin -*ulus*, which is a diminutive in masculine-form.

Evaluation: Clearly intended as an adjectival name, so it is corrected herein to feminine *rhombobula*.

Type locality: Dongshan, Fujian Province, China

Geolocation: 23.6689°, 117.3969° (map estimate).

WoRMS: 882579

Synonyms: No subjective synonyms.

***Hydroides rostrata* Pillai, 1971 (original name, junior homonym, replacement name *Hydroides gottfriedi* nomen novum)**

Status: Previously unreplaced junior homonym preoccupied by the invalid *Hydroides rostrata* Iroso, 1921, which was a *n. nom*. for the specimen of *Eupomatus uncinatus* non Philippi, *sensu* Ehlers, 1887, but a junior objective synonym of *Hydroides floridana* (Bush, 1910) as Bush had already re-named it. Replaced by *Hydroides gottfriedi* **nom. n.** here.

Etymology: Not stated, but *Hydroides rostrata* is likely named after the large rostrum-like verticil spine figured by the author. The adjective *rostratus* -*a* -*um*, means having a beak. The genitive replacement name *Hydroides gottfriedi* is in memory of Telesphore Gottfried Pillai (1930–2013), the original-name author.

Evaluation: Gender-variable adjective with correct original feminine ending. Usages as ‘*rostratus*’ exist (e.g., ten Hove and Kupriyanova 2009: 54).

Type locality: Hikkaduwa, Sri Lanka. Types were collected at both Hikkaduwa and Wellawatte. These localities are separated by some considerable distance, but the holotype at the Natural History Museum, London BM 1968–148, is from Hikkaduwa.

Geolocation: 6.1324°, 80.1000° (map estimate).

WoRMS: 328469

Synonyms: No subjective synonyms.

***Hydroides salazarvallejoi* Bastida-Zavala & ten Hove, 2002 (original binomen)**

Etymology: The authors named *Hydroides salazarvallejoi* as a dedication to Sergio Salazar-Vallejo.

Evaluation: Invariant genitive form *salazarvallejoi* from personal name Salazar-Vallejo.

Type locality: Cabo de la Aguja, Santa Marta region, Colombia, Caribbean Sea.

Geolocation: 11.3040°, -74.1937° (map estimate).

WoRMS: 328470

Synonyms: No subjective synonyms.

***Hydroides sanctaecrucis* Krøyer [in] Mörch, 1863 (originally Hydroides (Eucarphus) sanctae crucis)**

Etymology: Not stated, but *Hydroides sanctaecrucis* is clearly named after its type locality, Saint Croix Island, and the syntypes at the Zoological Museum, University of Copenhagen are labelled “Kr. St. Croix, legit Oerstedt”. The genitive of the feminine Latin noun *crux* ‘cross’ is *crucis*.

Evaluation: Place-name translated into Latin. The genitive-case noun *sanctaecrucis* is invariant.

Type locality: Saint Croix (unspecified further), Virgin Islands, Caribbean Sea.

Geolocation: 17.6949°, -64.7416° (map estimate for the port area).

WoRMS: 333645

Synonyms: Hydroides (Eupomatus) dianthoides Augener, 1922 [*partim*, *fide* Bastida-Zavala and ten Hove 2002: 147] (Haiti, Caribbean Sea)

***Hydroides similis* (Treadwell, 1929) (originally as *Eupomatus similis*)**

Etymology: Not stated, but an instance of the Latin adjective *similis* ‘similar to’. Later in the same work Treadwell (1929: 12) considered his *Hydroides californicus* (now *Hydroides crucigera*) as similar to his *Eupomatus similis*, which isn’t compared to any taxon, so the more logical application of the names would have been in reverse.

Evaluation: Masculine/feminine invariant adjective (*similis* -*e*) (Stearn 1983: 94).

Type locality: Unspecified beyond a “Lower California” location on label (Baja California, Mexico). The collector was Townsend, on the ‘Albatross’ voyage of 1911, and the location is perhaps more likely the Gulf of California than off the Pacific coast. Gulf coast sites mentioned by Treadwell where other polychaetes were collected include Isla Carmen and Isla San José, but there are many other possibilities (see Townsend 1916: 399, end map).

Geolocation: Unknown (30°, -115° (gazetteer) as Baja California general region, but perhaps inner coast).

WoRMS: 369244

Synonyms: No subjective synonyms.

***Hydroides similoides* Bastida-Zavala & ten Hove, 2002 (original binomen)**

Etymology: The authors state they named *Hydroides similoides* for its resemblance to *Hydroides similis* (type locality Baja California) thus combining the Latin adjective *similis* ‘similar to’ with the suffix -*oides*, also ‘similar to’.

Evaluation: Invariant adjectival suffix -*oides*.

Type locality: La Parguera (jetty of marine institute), Isla Magueyes, Puerto Rico.

Geolocation: 17.9700°, -67.0463° (map estimate).

WoRMS: 328471

Synonyms: No subjective synonyms.

***Hydroides simplidentata* Pillai, 2009 (originally as *Hydroides simplidentatus*)**

Etymology: The author states the name *Hydroides simplidentatus* “refers to the simple unmodified spines at the base of the enlarged coronal [verticil] spine”, combining Latin adjectives *simplus* -*a* -*um* ‘simple’ and *dentatus* -*a* -*um* ‘toothed’.

Evaluation: Corrected to the feminine form *simplidentata* in Sun et al. (2015: 79) as clearly an adjectival name.

Type locality: Unnamed reef north-west of Buffon Island (but cf. author’s supplied geolocation which is non-reef and east of Buffon Island), Kimberley, Western Australia.

Geolocation: -14.9167°, 124.8° (author as stated, but likely displaced incorrectly by ~13 km to the East).

WoRMS: 882648

Synonyms: No subjective synonyms.

***Hydroides sinensis* Zibrowius, 1972a (original binomen)**

Etymology: Not stated, but *Hydroides sinensis* is evidently named for its occurrence on the coast of China.

Evaluation: Masculine/feminine invariant Latin adjective (‘*sinensis*’) referring to China, a non-Latin geographic name.

Type locality: Off Qingdao (Zibrowius as ‘Tsindao’), China coast, northern Yellow Sea.

Geolocation: 36.0565°, 120.38° (map estimate).

WoRMS: 328472

Synonyms: No subjective synonyms.

***Hydroides spongicola* Benedict, 1887 (original binomen)**

Etymology: Not stated but *Hydroides spongicola* is evidently named from English ‘sponge’ as stem *spongi*- combined with -*cola* ‘dweller’, because of its association as “frail calcareous tubes in living sponges”.

Evaluation: Invariant compound noun in apposition with -*cola* as a substantival suffix.

Type locality: West offshore from Venice, Florida, Gulf of Mexico, USA, 48 m.

Geolocation: 27.0667°, -83.3542° (as from author as 27°04'00"N, 83°21'15"W).

WoRMS: 338021

Synonyms: No subjective synonyms.

***Hydroides steinitzi* Ben-Eliahu, 1972 (original binomen)**

Etymology: The species *Hydroides steinitzi* is dedicated to Heinz Steinitz.

Evaluation: Invariant genitive form *steinitzi* from personal name Steinitz.

Type locality: Sinai bank of Little Bitter Lake, Suez Canal, opposite Al-Kabrit on Egyptian bank.

Geolocation: 30.2662°, 32.5066° (opposite Al-Kabrit, map estimate).

WoRMS: 131014

Synonyms: No subjective synonyms.

***Hydroides stoichadon* Zibrowius, 1971(original binomen)**

Etymology: Not stated, but *Hydroides stoichadon* is from Greek Στοιχαδας (Stoichadas), an old name for Îles d’Hyères, an archipelago of small islands near Toulon, Mediterranean coast of France (H. Zibrowius pers. comm.).

Evaluation: Invariant Latinization created from Greek place-name, having the form of a noun in apposition.

Type locality: Cap du Merlan, the south west corner of Parc Nacional de Port Cros (island), off the Mediterranean coast of France.

Geolocation: 42.9960°, 6.3718° (map estimate).

WoRMS: 131015

Synonyms: No subjective synonyms.

***Hydroides tambalagamensis* Pillai, 1961 (original binomen)**

Etymology: Not stated but *Hydroides tambalagamensis* is evidently named after its place of collection, Tambalagam.

Evaluation: Masculine/feminine invariant ‘-*ensis*’ adjective created from a non-Latin place-name.

Type locality: Nachchikuda, Tambalagam Lake (a bay), eastern Sri Lanka.

Geolocation: 8.5333°, 81.1667° (map estimate).

WoRMS: 328474

Synonyms: *Hydroides spiculitubus* [noun in apposition] Pillai, 2009 (Long Reef, Kimberley, Western Australia)

***Hydroides tenhovei* Bastida-Zavala & de León González, 2002 (original binomen)**

Etymology: The authors dedicated the name *Hydroides tenhovei* to Harry ten Hove.

Evaluation: Invariant genitive form *tenhovei* from personal name ten Hove.

Type locality: Cabo San Lazaro, western coast of Baja California Sur, Mexico.

Geolocation: 24.7813°, -112.2905° (authors 24°50'N, 112°15'W, adjusted to be coastal).

WoRMS: 328475

Synonyms: No subjective synonyms.

***Hydroides trilobula* Chen & Wu, 1978 (originally *Hydroides trilobulus*)**

Etymology: Not stated, but the name for *Hydroides trilobulus* evidently refers to three vesicular verticil spines (three lobes), which are small ones, hence the diminutive Latin suffix -*ulus*.

Evaluation: Clearly meant as an adjectival name, so it is corrected herein to *trilobula*.

Type locality: Xisha Islands (Paracel Islands, unspecified further), South China Sea, of which Yongxing Island is the largest.

Geolocation: Imprecisely known (16.8833°, 112.2833° if Yongxing Island, map estimate).

WoRMS: 882593

Synonyms: No subjective synonyms.

***Hydroides trivesiculosa* Straughan, 1967b (originally *Hydroides trivesiculosus*)**

Etymology: Not stated, but the name for *Hydroides trivesiculosus* is evidently referring to the three lobes of the enlarged dorsal spine of the verticil, thus *tri* with Latin adjective *vesiculosus* -*a* -*um* ‘full of blisters’.

Evaluation: Gender-variable adjective with ending feminine as in Sun et al. (2015: 85) and Kupriyanova et al. (2015; 293), both mistakenly reporting the original name as ‘*trivesiculosa*’). Usages as ‘*trivesiculosus*’ (besides original) exist (e.g., ten Hove and Ben Eliahu 2005: 134).

Type locality: Heron Island, Queensland coast, Australia, collected close to the marine station by Dew (map in Straughan 1967b).

Geolocation: -23.4430°, 151.9110° (map estimate).

WoRMS: 882647

Synonyms: No subjective synonyms.

***Hydroides trompi* Bastida-Zavala & ten Hove, 2003 (original binomen)**

Etymology: The authors named *Hydroides trompi* dedicated to Jossy S. Tromp, a student of ten Hove.

Evaluation: Invariant genitive form *trompi* from personal name Tromp.

Type locality: Lower chamber wall and floor, Miraflores Locks, Panama Canal, Panama.

Geolocation: 8.9967, -79.5964 (authors).

WoRMS: 328478

Synonyms: No subjective synonyms.

***Hydroides tuberculata* Imajima, 1976a (original binomen)**

Etymology: Not stated, but the name for *Hydroides tuberculata* may refer to tubercles (knobs) on each verticil spine as the Latin noun *tuberculum* refers to a swelling or lump.

Evaluation: Gender-variable adjectival form of *tuberculum* with correct original feminine ending. Usages as ‘*tuberculatus*’ exist (e.g., Bailey-Brock 1987: 282).

Type locality: Urata (beach), Tanegashima (island), Southern Japan. Imajima (1976a) also gives records for Sumiyoshi, and off Nishinoomote Harbour, Tanegashima, but a Urata specimen is the holotype (NSMT-Pol. H-120) at the National Museum of Nature & Science, Tokyo.

Geolocation: 30.8233°, 131.0409° (map estimate, Urata).

WoRMS: 871950

Synonyms: No subjective synonyms.

***Hydroides uncinata* (Philippi, 1844) (originally as *Eupomatus uncinatus*)**

Status: Name now disused and representing a *species inquirenda* which is the type species of *Eupomatus*. It is not a candidate *nomen oblitum* (used in taxonomy as valid by Zibrowius 1968: 109, Hartman 1969: 757, Gibbs 1971: 202, Pillai 1972: 15, Day 1973: 132, and others), but the taxon it represents may be indeterminable unless original specimens are found. Zibrowius (1968) named *Hydroides pseudouncinata* to establish a separation from *Hydroides uncinata*, and Pillai (1972) did likewise with *Hydroides okudai*. The name does not obviously threaten the validity of subsequent names, but it is notable that several *Hydroides uncinata* usages have been assigned elsewhere, e.g., Serpula (Hydroides) uncinata non Philippi, *sensu* Gravier, 1906 to *Hydroides heterocera*.

Etymology: Not stated, but Philippi described the verticil spines as with “*cornubus octo, apice incurvo uncinatis*” (eight horns, curved tip hooked), thus the name refers to the hooked spines, from the Latin adjective *uncinatus* ‘hooked’.

Evaluation: Gender-variable adjective with correct feminine ending. Usages in *Hydroides* as ‘*uncinatus*’ exist (e.g., Ehlers 1913: 582).

Type locality: Unspecified Mediterranean, but can be narrowed to the Tyrrhenian Sea coast of Italy as Philippi’s activities were in western Italy, and plausibly to Naples as he was based there prior to 1844.

Geolocation: Unknown (Tyrrhenian Sea, with Naples shore (40.8327°, 14.2358° map estimate) a possible point location).

WoRMS: 156135

Synonyms: As *species inquirenda* has no synonyms.

***Hydroides uniformis* Imajima & ten Hove, 1986 (original binomen)**

Etymology: Not stated, but the name *Hydroides uniformis* evidently refers to the straight, thick, unornamented spines of the verticil which are described as “uniform”.

Evaluation: Masculine/feminine invariant adjective (*uniformis* -*e*) (Stearn 1983: 94).

Type locality: Kesao, Guadalcanal, Solomon Islands, Pacific Ocean.

Geolocation: -9.25°, 159.6667° (map estimate).

WoRMS: 369245

Synonyms: No subjective synonyms.

***Hydroides vizagensis* Lakshmana Rao, 1969 (original binomen)**

Etymology: Not stated, but the name *Hydroides vizagensis* likely derives from the collection location, Visakhapatnam, which has the nickname Vizag.

Evaluation: Masculine/feminine invariant ‘-*ensis*’ adjective created from a non-Latin place-name.

Type locality: Naval Base (collected off settlement panels), Visakhapatnam Harbour, east coast of India, Bay of Bengal.

Geolocation: 17.6938°, 83.2739° (map estimate).

WoRMS: 870503

Synonyms: No subjective synonyms.

***Hydroides xishaensis* Chen & Wu, 1978 (original binomen)**

Etymology: Not stated, but *Hydroides xishaensis* is evidently named after its area of collection, the Xisha Islands.

Evaluation: Masculine/feminine invariant ‘-*ensis*’ adjective created from a non-Latin geographic name.

Type locality: Xisha Islands (Paracel Islands, unspecified further), South China Sea, of which Yongxing Island is the largest.

Geolocation: Imprecisely known (16.8833°, 112.2833° if from Yongxing Island, map estimate).

WoRMS: 328480

Synonyms: No subjective synonyms.

## Discussion

### Name characteristics and potential variation

Place-names (23), and personal names (16) make up more than a third (36%) of the 107 non-synonymised species-group names in *Hydroides*, with most of the remainder (68) being descriptive of species character states, with a remarkable number relating to operculum morphology (54). The (perhaps) ‘small-eared’ *Hydroides microtis* was the only species where the reason for the author’s choice was not obvious. Otherwise three species were named for their attractive appearance (*Hydroides dianthus*, *Hydroides elegans*, *Hydroides elegantula*), three were named for the animals they were found on (*Hydroides fusicola*, *Hydroides protulicola*, *Hydroides spongicola*), four names are comparative (*Hydroides affinis*, *Hydroides pseudouncinata*, *Hydroides similis*, *Hydroides similoides*), two species names probably relate to body size (*Hydroides humilis*, *Hydroides parva*), and one species name relates to the tube form (*Hydroides hexagona*).

Currently the 107 names include 41 which should be gender invariant (including 17 nouns in apposition, including two acronyms), and 23 with adjectival masculine/feminine endings in -*is*, which would only change (to -*e*) if moved to a neuter genus (13 of these are place-names). The remaining 43 names are fully gender variable. There are 68 adjectival names in total (including 19 adjectival place-names), with only two adjectival names completely invariant.

### Type locality distribution

**Figure 1.**
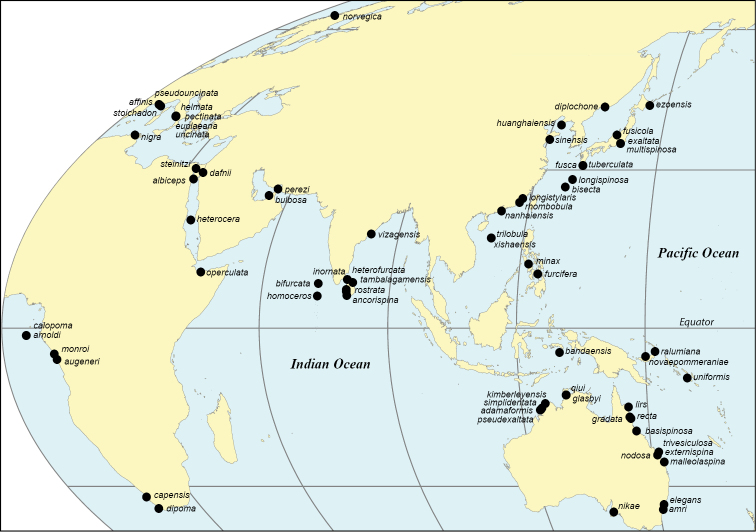
*Hydroides* species type localities of the Eastern Hemisphere (Indian Ocean and Western Pacific). Labels are current species-group names (except homonym *Hydroides rostrata* renamed herein as *Hydroides gottfriedi* nom. n.)

**Figure 2.**
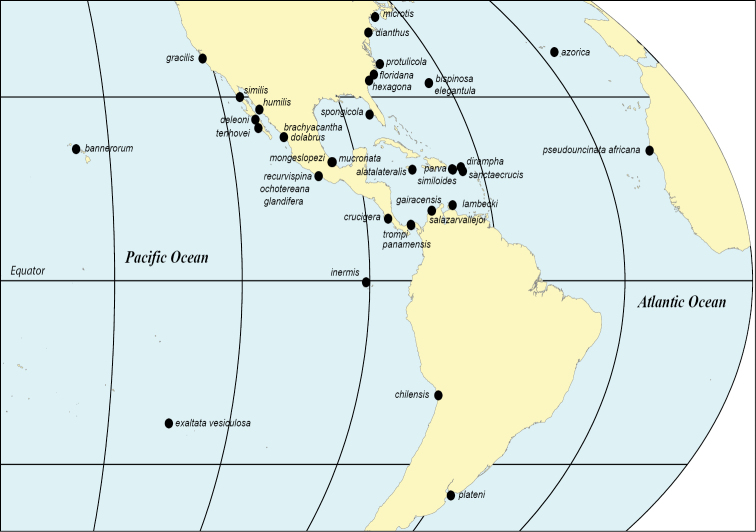
*Hydroides* species type localities of the Western Hemisphere (Americas and Eastern Pacific). Labels are current species-group names. Map grids 30° intervals.

Type localities of the *Hydroides* serpulids listed are, with one exception, in shallow-water coastal locations in temperate to tropical waters between 43.3°N and 35.3°S (Figs 1–2). *Hydroides norvegica* is the exception from deeper water (but still inshore), and occurred at the highest latitude at 63.4°N. It is the most cold tolerant based on type locality, with a 20° latitudinal gap to all other species type localities, although its distribution extends south into the Mediterranean (Zibrowius 1971). The western Pacific Ocean (Australia to northern Japan) has the biggest group of new species at 39, with another 15 species in the northern Indian Ocean and Red Sea (Fig. 1), a total of 54 for the greater Indo-Pacific. The Americas north of the equator have 13 new species on the East Pacific coast, and 18 on the Western Atlantic coast and the Caribbean/Gulf of Mexico area (Fig. 2), a total of 31 for North American and Caribbean coasts combined. Europe (including Azores) has only 10 new species, mostly in the Mediterranean Sea (Fig. 1). This leaves only 12 other species described from elsewhere. Notably, few new species (9) have been reported from the major continental coasts of the South American coast south of the equator, and the African coast (outside of the Red Sea and Mediterranean, and including Madagascar), but this may be partly a reflection of lesser sampling effort, and also the consequence of other areas being examined first, given that *Hydroides* species are readily translocated on the floating objects and vessel hulls they colonise.

## Supplementary materials

Supplementary material 1
Hydroides type-species data summary Data type: spreadsheet Explanation note: Name categories, original-name WoRMS links, geolocations, locations, etc. This dataset is made available under the Open Database License (http://opendatacommons.org/licenses/odbl/1.0/). The Open Database License (ODbL) is a license agreement intended to allow users to freely share, modify, and use this Dataset while maintaining this same freedom for others, provided that the original source and author(s) are credited.Geoffrey B. Read, Harry A. ten Hove, Yanan Sun, Elena K. Kupriyanova

## References

[B1] Amoureux L Rullier F Fishelson L (1978) Systématique et Écologie d’Annélides Polychètes de la presqu’îl du Sinai. Israel Journal of Zoology 27(2–3): 57–163.

[B2] Augener H (1918) Polychaeta Beiträge zur Kenntnis der Meeresfauna Westafrikas 2(2): 67–625. http://biodiversitylibrary.org/page/7172280

[B3] Augener H (1922) Über litorale polychaeten von Westindien. Sitzungsberichte der Naturforschender Freunde zu Berlin 1922(3–5): 38–53. http://biodiversitylibrary.org/page/43656381

[B4] Augener H (1925) Die Polychaeten der Südsee-Expedition der Hamburgischen wissenschaftliche Stiftung 1908–1909. Mitteilungen der Zoologisches Staatsinstitut und zoologisches Museum, Hamburg 41: 53–70.

[B5] Augener H (1927) Polychaeten von Neu-Pommern. Sitzungsberichte der Gesellschaft der naturforschende Freunde zu Berlin 1926: 119–152.

[B6] Augener H (1934) Polychaeten aus den Zoologischen Museen von Leiden und Amsterdam. IV (Schluss). Zoölogische Mededeelingen Rijks Museum van Natuurlijke Historie Leiden 17: 67–160. http://www.repository.naturalis.nl/record/318471

[B7] Bailey-Brock JH (1987) The polychaetes of Fanga’uta Lagoon and coral reefs of Tongatapu, Tonga, with discussion of the Serpulidae and Spirorbidae. Bulletin of the Biological Society of Washington 7: 280–294.

[B8] Bailey-Brock JH (1991) Tubeworms (Serpulidae, Polychaeta) collected from sewage outfalls, coral reefs and deep waters off the Hawaiian Islands, including a new *Hydroides* species. Bulletin of Marine Science 48(2): 198–207. http://www.ingentaconnect.com/contentone/umrsmas/bullmar/1991/00000048/00000002/art00006

[B9] Bastida-Zavala JR ten Hove HA (2002) Revision of *Hydroides* Gunnerus, 1768 (Polychaeta: Serpulidae) from the Western Atlantic Region. Beaufortia 52(9): 103–178.

[B10] Bastida-Zavala JR ten Hove HA (2003) Revision of *Hydroides* Gunnerus, 1768 (Polychaeta: Serpulidae) from the Eastern Pacific Region and Hawaii. Beaufortia 53(4): 67–110.

[B11] Bastida-Zavala JR de León González JA (2002) A new species of *Hydroides* (Polychaeta: Serpulidae) from western Mexico. Journal of the Marine Biological Association of the United Kingdom 82(3): 389–393. doi: 10.1017/S0025315402005623

[B12] Bean W (1844) A supplement of new species. In: Thorpe C. British Marine Conchology, Edward Lumley, London, 263–267. doi: 10.5962/bhl.title.11208

[B13] Bellan G (2001) Polychaeta In: Costello MJ et al. (Eds) European register of marine species: a check-list of the marine species in Europe and a bibliography of guides to their identification. Collection Patrimoines Naturels 50: 214–231. http://www.vliz.be/imisdocs/publications/77636.pdf

[B14] Benedict JE (1887) Descriptions of ten species and one new genus of annelids from the dredgings of the U.S. Fish Commission Steamer Albatross. Proceedings of the United States National Museum 9: 547–553. doi: 10.5479/si.00963801.9-594.547

[B15] Ben-Eliahu MN (1972) A description of *Hydroides steinitzi* n. sp. (Polychaeta: Serpulidae) from the Suez Canal with remarks on the serpulid fauna of the Canal. Israel Journal of Zoology 21(2): 77–81. http://www.tandfonline.com/doi/abs/10.1080/00212210.1972.10688351

[B16] Ben-Eliahu MN Hove HA ten (1992) Serpulids, Annelida: Polychaeta, along the Mediterranean coast of Israel—New population build-ups of Lessepsian migrants. Israel Journal of Zoology 38(1): 35–53. http://www.tandfonline.com/doi/abs/10.1080/00212210.1992.10688664

[B17] Ben-Eliahu MN Hove HA ten (2011) Serpulidae (Annelida: Polychaeta) from the Suez Canal—From a Lessepsian Migration Perspective. Zootaxa 2848 (Monograph): 1–147. http://www.mapress.com/zootaxa/list/2011/2848.html

[B18] Billard A (1907) Hydroïdes de la Collection Lamarck du Muséum de Paris. I Plumulariidae Annales des sciences naturelles. Zoologie (Series 9) 5: 319–335. http://biodiversitylibrary.org/page/34908193

[B19] Bosc LAG (1802) Histoire naturelles des vers, contenant leur description et leurs moeurs; avec figures dessinées d’après nature. Tome Premiere. De l’imprimerie de Guilleminet, chez Deterville, Paris, 324 pp. doi: 10.5962/bhl.title.64025

[B20] Brown RW (1956) Composition of scientific words. Reprint edition. Smithsonian Institution Press, Washington, 882 pp.

[B21] Bush KJ (1905) Tubicolous annelids of the tribes Sabellides and Serpulides from the Pacific Ocean. Harriman Alaska Expedition 12: 169–346. doi: 10.5962/bhl.title.27846

[B22] Bush KJ (1910) Description of new serpulids from Bermuda with notes on known forms from adjacent regions. Proceedings of the Academy of Natural Sciences, Philadelphia 62: 490–501. http://biodiversitylibrary.org/page/26294605

[B23] Chamberlin RV (1919) New polychaetous annelids from Laguna Beach, California. Journal of Entomology and Zoology. Pomona College 11(1): 1–23. http://biodiversitylibrary.org/page/12263520

[B24] Chen M Wu BL (1978) Two new species of the genus *Hydroides* (Polychaeta, Serpulidae) from the Xisha Islands, Guandong Province, China. Studia Marina Sinica 12: 141–145.

[B25] Chen M Wu BL (1980) Two new species of the genus *Hydroides* (Polychaeta, Serpulidae). Oceanologia et Limnologia Sinica 11(3): 247–250.

[B26] Claparède E (1870a) Les Annélides Chétopodes du Golfe de Naples. Seconde partie. Ordre IIme. Annélides Sédentaires. Mémoires de la Société de physique et d’histoire naturelle de Geneve 20(1): 1–225. http://biodiversitylibrary.org/page/2071623

[B27] Claparède E (1870b) Les Annélides Chétopodes du Golfe de Naples. Supplement. Mémoires de la Société de physique et d’histoire naturelle de Geneve 20(2): 365–542. http://biodiversitylibrary.org/page/2094031

[B28] Colwin AL Colwin LH (1961) Fine structure of the spermatozoon of *Hydroides hexagonus* (Annelida), with special reference to the acrosomal region. Journal of Biophysical and Biochemical Cytology 10(2): 211–230. doi: 10.1083/jcb.10.2.211 10.1083/jcb.10.2.211 PMC2225072 13694876

[B29] Costello DP Davidson ME Eggers A Fox MH Henley C (1957) Methods for Obtaining and Handling Marine Eggs and Embryos. Marine Biological Laboratory & Lancaster Press, Woods Hole, Massachusetts, 247 pp. doi: 10.5962/bhl.title.1023

[B30] David N Gosselin M (2002) Gender agreement of avian species names. Bulletin of the British Ornithologists’ Club 122: 14–49.

[B31] Day JH (1951) The Polychaet [sic] Fauna of South Africa. Part I. The intertidal and estuarine Polychaeta of Natal and Mosambique. Annals of the Natal Museum 12(1): 1–67. http://content.ajarchive.org/cdm4/document.php?CISOROOT=/03040798&CISOPTR=905&REC=12

[B32] Day JH (1967) A monograph on the Polychaeta of Southern Africa. Part 2. Sedentaria. Trustees of the British Museum (Natural History) London, 459–878. doi: 10.5962/bhl.title.8596

[B33] Day JH (1973) New Polychaeta from Beaufort, with a key to all species recorded from North Carolina. National Oceanic and Atmospheric Administration Technical Report NMFS CIRC-375: 1–140. doi: 10.5962/bhl.title.62852

[B34] Delle Chiaje S (1828) Memorie sulla storia e notomia degli animali senza vertebre del Regno di Napoli. vol. 3. Stamperia della Societa' Tipografica, Napoli, 232 pp. [plates published separately in 1830, dated “1822”] http://www.biodiversitylibrary.org/item/40491

[B35] Dew B (1959) Serpulidae (Polychaeta) from Australia. Records of the Australian Museum 25: 19–56. doi: 10.3853/j.0067-1975.25.1959.654

[B36] Ehlers E (1887) Reports on the results of dredging, under the direction of L. F. Pourtalès, during the years 1868–1870, and of Alexander Agassiz, in the Gulf of Mexico (1877–78), and in the Caribbean Sea (1878–79), in the U.S. Coast Survey steamer “Blake”, Lieut-Com. C. D. Sigsbee, U.S.N. and Commander J. R. Bartlett, U.S.N., commanding. XXXI. Report on the Annelids. Memoirs of the Museum of Comparative Zoology, Harvard 15: 1–335. doi: 10.5962/bhl.title.65639

[B37] Ehlers E (1913) Die Polychaeten-Sammlungen der Deutschen Südpolar-Expedition 1901–1903. Deutsche Südpolar-Expedition 13(Zoologie V): 397–598. http://biodiversitylibrary.org/page/2139283

[B38] Fauvel P (1918) Annélides polychètes des côtes d’Arabie récoltées par M. Ch. Pérez. Bulletin du Muséum d’Histoire Naturelle, Paris 24(5): 329–344. http://biodiversitylibrary.org/page/5037658

[B39] Fauvel P (1919) Annélides polychètes des îles Gambier et Touamotou. Bulletin du Muséum National d’Histoire Naturelle, Paris 25(5): 336–343. http://biodiversitylibrary.org/page/5027005

[B40] Fischli H (1903) Polychäten von Ternate. Senckenbergische Naturforschende Gesellschaft Abhandlungen 25(1): 90–136. http://biodiversitylibrary.org/page/25232322

[B41] Frauenfeld G von (1855) Naturhistorische Fragmente: gesammelt auf einer Reise am Rothen Meere im Frühjahre 1855. Sitzungsberichte der Mathematisch-Naturwissenschaftlichen Classe der Kaiserlichen Akademie der Wissenschaften (Wien) 18: 66–87. http://biodiversitylibrary.org/page/6441585

[B42] Gibbs PE (1971) The polychaete fauna of the Solomon Islands. Bulletin of the British Museum (Natural History) Zoology 21(5): 101–211. doi: 10.5962/bhl.part.10154

[B43] Grave BH (1937) *Hydroides hexagonus*. In: Galtsoff PS, Lutz FE, Welch PS, Needham JG (Eds) Culture methods for invertebrate animals. Comstock Publishing Company, Ithaca, New York, 185–187. doi: 10.5962/bhl.title.6012

[B44] Grave BH Oliphant JF (1930) The longevity of unfertilized gametes. Biological Bulletin (Woods Hole) 59(3): 233–239. doi: 10.2307/1536993

[B45] Gravier C (1906) Sur les Annélides Polychètes de la Mer Rouge (Serpulides). Bulletin du Muséum d’Histoire Naturelle, Paris 12: 110–115. http://biodiversitylibrary.org/page/5021287

[B46] Grube AE (1868) Beschreibungen einiger von Georg Ritter von Frauenfeld gesammelter Anneliden und Gephyreen des rothen Meeres. Verhandlungen der kaiserlich-königlichen zoologisch-botanischen Gesellschaft in Wien 18: 629–650. http://biodiversitylibrary.org/page/25254604

[B47] Grube AE (1870) Beschreibungen neuer oder weniger bekannter von Hrn. Ehrenberg gesammelter Anneliden des rothen Meeres. Monatsbericht der Koniglich Preussischer Akademie der Wissenschaften zu Berlin 1869: 484–521. http://www.biodiversitylibrary.org/page/36276705

[B48] Grube AE (1878a) Einige neue anneliden aus Japan. Jahres-Bericht der Schlesischen Gesellschaft für Vaterländische Cultur 55 [1878 for 1877 year]: 104–106. http://biodiversitylibrary.org/page/37206819

[B49] Grube AE (1878b) Annulata Semperiana. Beiträge zur Kenntniss der Annelidenfauna der Philippinen. Memoires de L’Académie Impériale.des Sciences de St.-Pétersbourg VII Série 25(8): 1–300. doi: 10.5962/bhl.title.85345

[B50] Gunnerus JE (1768) Om nogle Norske Coraller. Det Kongelige Norske Videnskabernes Selskabs Skrifter 4: 38–73. http://gdz.sub.uni-goettingen.de/dms/load/img/?PPN=PPN481641912_0004&IDDOC=273812

[B51] Hadfield MG (1998) The D P Wilson lecture. Research on settlement and metamorphosis of marine invertebrate larvae: past, present and future. Biofouling 12(1–3): 9–29. doi: 10.1080/08927019809378343

[B52] Harper D (2001–2016) . Online Etymology Dictionary. http://www.etymonline.com/index.php

[B53] Hartman O (1956) Polychaetous annelids erected by Treadwell, 1891 to 1948, together with a brief chronology. Bulletin of the American Museum of Natural History 109(2): 239–310. http://hdl.handle.net/2246/1145

[B54] Hartman O (1959) Catalogue of the polychaetous annelids of the world. Parts 1 & 2. Allan Hancock Foundation Occasional Paper 23: 1–628

[B55] Hartman O (1965) Supplement and index to the catalogue of the polychaetous annelids of the World, including additions and emendations since 1959. Allan Hancock Foundation Occasional Paper 23 Supplement and Index: 1–197. http://digitallibrary.usc.edu/cdm/ref/collection/p15799coll82/id/19369

[B56] Hartman O (1969) Atlas of the sedentariate polychaetous annelids from California. Allan Hancock Foundation, University of Southern California, 812 pp.

[B57] Hartmann-Schröder G (1962) Die Polychaeten des Eulitorals. In: Hartmann-Schröder G and Hartmann G. Zur Kenntnis des Eulitorals der chilenischen Pazifikküste und der argentinischen Küste Südpatagoniens unter besonderer Berücksichtigung der Polychaeten und Ostracoden. Mitteilungen aus dem Hamburgischen zoologischen Museum und Institut 60: 57–270.

[B58] Haswell WA (1883) On some new Australian tubicolous annelids. Proceedings of the Linnean Society of New South Wales 7: 633–638. http://biodiversitylibrary.org/page/6462370

[B59] Hove HA ten (1990) Description of *Hydroides bulbosus* sp. n. (Polychaeta, Serpulidae), from the Iranian Gulf, with a terminology for opercula of *Hydroides*. Beaufortia 41(16): 115–120.

[B60] Hove HA ten Ben-Eliahu MN (2005) On the identity of *Hydroides priscus* Pillai 1971—Taxonomic confusion due to ontogeny in some serpulid genera (Annelida: Polychaeta: Serpulidae). Senckenbergiana Biologica 85(2): 127–145.

[B61] Hove HA ten Kupriyanova EK (2009) Taxonomy of Serpulidae (Annelida, Polychaeta): The state of affairs. Zootaxa 2036: 1–125. http://www.mapress.com/j/zt/issue/view/2173

[B62] Imajima M (1976a) Serpulid polychaetes from Tanega-shima, southwest Japan. Memoirs of the National Science Museum 9: 123–143. http://ci.nii.ac.jp/naid/110004313275

[B63] Imajima M (1976b) Serpulinae (Annelida, Polychaeta) from Japan I. the genus *Hydroides* Bulletin of the National Science Museum, Tokyo, Series A (Zoology) 2(4): 229–248. http://ci.nii.ac.jp/naid/40005325183

[B64] Imajima M (1982) Serpulinae (Polychaetous Annelids) from the Palau and Yap Islands, Micronesia. Proceedings of the Japanese Society of Systematic Zoology 23: 37–55. http://ci.nii.ac.jp/naid/110002339223

[B65] Imajima M ten Hove HA (1986) Serpulinae (Annelida, Polychaeta) from Nauru, the Gilbert Islands (Kiribati) and the Solomon Islands. Proceedings of the Japanese Society of Systematic Zoology 32: 1–16. http://ci.nii.ac.jp/naid/110002339356

[B66] Imajima M ten Hove HA (1989) Two new species of serpulids (Annelida, Polychaeta) from Sesoko Island, Okinawa. Bulletin of the National Science Museum, Tokyo, Series A (Zoology) 15(1): 11–17. http://ci.nii.ac.jp/naid/110004311713

[B67] International Commission on Zoological Nomenclature [ICZN] (1961) International Code of Zoological Nomenclature, adopted by the XV International congress of Zoology. The International Trust for Zoological Nomenclature, London, 176 pp. doi: 10.5962/bhl.title.50303

[B68] ICZN (1985) International Code of Zoological Nomenclature Third Edition adopted by the XX General Assembly of the International Union of Biological Sciences. The International Trust for Zoological Nomenclature, London, 338 pp. doi: 10.5962/bhl.title.50611

[B69] ICZN (1999) International Code of Zoological Nomenclature Fourth edition adopted by the International Union of Biological Sciences. The International Trust for Zoological Nomenclature, London, 306 pp. doi: 10.5962/bhl.title.50608

[B70] Iroso I (1921) Revisione dei Serpulidi e Sabellidi del Golfo di Napoli. Pubblicazioni della Stazione Zoologica di Napoli 3: 47–91.

[B71] Jones ML (1962) On some polychaetous annelids from Jamaica, the West Indies. Bulletin of the American Museum of Natural History 124(5): 169–212. http://hdl.handle.net/2246/1213

[B72] Kinberg JGH (1867 (or late 1866)) Annulata nova. [Continuatio.]. Öfversigt af Kongl. [sic] Vetenskapsakademiens förhandlingar, Stockholm 23(9): 337–357. http://biodiversitylibrary.org/page/32287795

[B73] Kupriyanova EK Sun Y Hove HA ten Wong E Rouse GW (2015) Serpulidae (Annelida) of Lizard Island, Australia. Zootaxa 4019(1): 275–353. doi: 10.11646/zootaxa.4019.1.13 10.11646/zootaxa.4019.1.13 26624073

[B74] Lakshmana Rao MV (1969) Fouling serpulids from some Indian harbours. Journal of the Timber Development Association of India 15(2): 1–20.

[B75] de León González JA (1990) Dos serpúlidos nuevos para el Pacifico Mexicano y duplicidad opercular en *Hydroides crucigerus* [sic] (Polychaeta: Serpulidae). Revista de biologia tropical 38(2A): 335–338. http://revistas.ucr.ac.cr/index.php/rbt/article/view/25383

[B76] Lewis CT Short C (1891) Harpers Latin dictionary. A new Latin dictionary: founded on the translation of Freund’s Latin-German lexicon Harper, New York, 2019 pp. https://archive.org/details/LewisAndShortANewLatinDictionary

[B77] Marenzeller E von (1885) Südjapanische Anneliden. II. Ampharetea, Terebellacea, Sabellacea, Serpulacea Denkschriften der Akademie der Wissenschaften Mathematisch-Naturwissenschaftliche Classe. Wien. 49(2): 197–224. http://biodiversitylibrary.org/page/7102598

[B78] Marion AF (1875) Sur les Annélides de Marseille. Revue des sciences naturelle. Montpellier 4(1): 301–312. http://biodiversitylibrary.org/page/14526461

[B79] Marion AF Bobretzky N (1875) Étude des Annélides du golfe de Marseille. Annales des Sciences Naturelles, Paris (series 6) 2: 1–106. http://biodiversitylibrary.org/page/33155516

[B80] McIntosh WC (1923) A monograph of the British marine annelids. Volume IV Part II. Polychaeta Sabellidae to Serpulidae with additions to the British marine Polychaeta during the publication of the monograph. Ray Society 4(2): 251–538.

[B81] Moen TL (2006) A translation of Bishop Gunnerus’ description of the species *Hydroides norvegicus* with comments on his *Serpula triqvetra*. Scientia Marina 70 (Supplement 3): 115–123. doi: 10.3989/scimar.2006.70s3115

[B82] Monro CCA (1933) The Polychaeta Sedentaria collected by Dr. C. Crossland at Colón, in the Panama Region, and the Galapagos Islands during the Expedition of the S.Y. ‘St. George’. Proceedings of the Zoological Society of London 103(4): 1039–1092. doi: 10.1111/j.1096-3642.1933.tb01640.x

[B83] Monro CCA (1937) Polychaeta The John Murray Expedition 1933–1934 Scientific Reports. 4(8): 243–321. http://biodiversitylibrary.org/page/49518757

[B84] Monro CCA (1938) On a new species of serpulid polychate from the Shoreham Harbour Canal, Sussex. Annals and Magazine of Natural History (Series 11) 1(1): 73–78. doi: 10.1080/00222933808526742

[B85] Mörch OAL (1863) Revisio critica Serpulidarum. Et Bidrag til Rørormenes Naturhistorie. Naturhistorisk Tidsskrift, København, Ser. 3, 1: 347–470. http://www.archive.org/details/naturhistoriskti01copeuoft

[B86] Murray A Hutchings P Pillai TG (2010) Note on *Hydroides malleolaspinus* from the Kimberleys of Western Australia (Polychaeta: Serpulidae). Records of the Australian Museum 62(3): 393–394. doi: 10.3853/j.0067-1975.62.2010.1564

[B87] Okuda S (1934) Some tubicolous annelids from Hokkaido. Journal of the Faculty of Science, Hokkaido University, Series 6, Zoology 3(4): 233–246. http://hdl.handle.net/2115/26971

[B88] Okuda S (1937) Annelida polychaeta in Onagawa Bay and its vicinity. I. Polychaeta Sedentaria. Science Reports of the Tohoku Imperial University. Fourth Series Biology 12(1): 45–69.

[B89] Philippi A (1844) Einige Bemerkungen über die Gattung *Serpula*, nebst Aufzählung der von mir im Mittelmeer mit dem Thier beobachteten Arten. Archiv für Naturgeschichte Berlin 10(1): 186–198. doi: 10.5962/bhl.part.29558

[B90] Pillai TG (1960) Some marine and brackish water serpulid polychaeta from Ceylon, including new genera and species. Ceylon Journal of Science (Biological Sciences) 3(1): 1–40.

[B91] Pillai TG (1961) Annelida Polychaeta of Tambalagam Lake, Ceylon. Ceylon Journal of Science (Biological Sciences) 4(1): 1–40. http://dl.nsf.ac.lk/handle/1/7600

[B92] Pillai TG (1965) Annelida Polychaeta from the Philippines and Indonesia. Ceylon Journal of Science (Biological Sciences) 5(2): 112–177. http://dl.nsf.ac.lk/handle/1/7733

[B93] Pillai TG (1971) Studies on a collection of marine and brackish-water polychaete annelids of the family Serpulidae from Ceylon. Ceylon Journal of Science (Biological Sciences) 9(2): 88–130.

[B94] Pillai TG (1972) A review and revision of the systematics of the genera *Hydroides* and *Eupomatus* together with an account of their phylogeny and zoogeography. Ceylon Journal of Science (Biological Sciences) 10(1): 7–31. http://dl.nsf.ac.lk/handle/1/7815

[B95] Pillai TG (2009) Descriptions of new serpulid polychaetes from the Kimberleys of Australia and discussion of Australian and Indo-West Pacific species of *Spirobranchus* and superficially similar taxa. Records of the Australian Museum 61(2): 93–199. doi: 10.3853/j.0067-1975.61.2009.1489

[B96] Pixell HLM (1913) Polychaeta of the Indian Ocean, together with some species from the Cape Verde Islands. The Serpulidae, with a classification of the genera *Hydroides* and *Eupomatus*. Transactions of the Linnean Society of London, Series 2 16: 69–92. doi: 10.1111/j.1096-3642.1914.tb00125.x

[B97] Pratt HS (1916) A manual of the common invertebrate animals, excluding insects. A. C. McClurg and Co., Chicago, 737 pp. doi: 10.5962/bhl.title.1245

[B98] Read GB Fauchald K (Ed.) (2016) World Polychaeta database. http://www.marinespecies.org/polychaeta

[B99] Read GB Hove HA ten (2016) *Hydroides* Gunnerus, 1768. http://www.marinespecies.org/polychaeta/aphia.php?p=taxdetails&id=129566

[B100] Ribas J Hutchings PA (2015) Lizard Island Polychaete Workshop: sampling sites and a checklist of polychaetes. Zootaxa 4019(1): 7–34. doi: 10.11646/zootaxa.4019.1.4 10.11646/zootaxa.4019.1.4 26624064

[B101] Rioja E (1941a) Estudios Anelidologicos. II. Observaciones acerca de varias especies del genero *Hydroides* Gunnerus (sensu Fauvel) de las costas Mexicanas del Pacifico. Anales del Instituto de Biologia (Mexico) 12(1): 161–175.

[B102] Rioja E (1941b) Estudios Anelidologicos. III. Datos para el conocimiento de la fauna de poliquetos de las costas del pacifico de Mexico. Anales del Instituto de Biologia, Mexico 12(2): 669–746.

[B103] Rioja E (1942) Estudios Anelidologicos. IV. Observaciones sobre especies de serpulidos de las costas del Pacifico de Mexico, con descripcion de una especie nueva del genero *Hydroides*. Anales del Instituto de Biologia (Mexico) 13(1): 125–135.

[B104] Rioja E (1958) Estudios Anelidologicos. XXI. Observaciones acerca de algunas especies de serpulidos de los generos *Hydroides* y *Eupomatus* de las costas Mexicanas del Golfo de Mexico. Anales del Instituto de Biologia, Mexico 28(1/2): 247–266 [1957 issue]

[B105] Schmarda LK (1861) Neue wirbellose Thiere beobachtet und gesammelt auf einer Reise um die Erdr [sic] 1853 bis 1857 von Ludwig K. Schmarda. Erster Band. Turbellarien, Rotatorien und Anneliden. Zweite Hälfte. Wilhelm Engelmann, Leipzig, 164 pp. doi: 10.5962/bhl.title.14426

[B106] Stearn WT (1983) Botanical Latin. 3^rd^ edition revised. David & Charles, London, 566 pp.

[B107] Straughan D (1967a) Marine Serpulidae (Annelida: Polychaeta) of eastern Queensland and New South Wales. Australian Journal of Zoology 15(1): 201–261. doi: 10.1071/ZO9670201

[B108] Straughan D (1967b) Some Serpulidae (Annelida: Polychaeta) from Heron Island, Queensland. University of Queensland Papers [Great Barrier Reef Committee, Heron Island Research Station] 1(2): 27–45.

[B109] Sun R Yang D (2000) Study on *Hydroides* (Polychaeta: Serpulidae) from waters off China. I. Studia Marina Sinica 42: 116–135. [Chinese with English summary]

[B110] Sun R Yang D (2014) Annelida Polychaeta III Sabellida. Fauna Sinica Invertebrata 54: 1–493.

[B111] Sun Y Wong E Hove HA ten Hutchings PA Williamson JE Kupriyanova EK (2015) Revision of the genus *Hydroides* (Annelida: Serpulidae) from Australia. Zootaxa 4009(1): 1–99. doi: 10.11646/zootaxa.4009.1.1 10.11646/zootaxa.4009.1.1 26623840

[B112] Sun Y Wong E Tovar-Hernández MA Williamson JE Kupriyanova EK (2016) . Is *Hydroides brachyacantha* (Serpulidae : Annelida) a widespread species? Invertebrate Systematics 30(1): 41–59. doi: 10.1071/IS15015

[B113] Tovar-Hernández MA Villalobos-Guerrero TF Kupriyanova EK Sun Y (2016 [online 2015]). A new fouling *Hydroides* (Annelida, Sabellida, Serpulidae) from southern Gulf of California. Journal of the Marine Biological Association of the United Kingdom 96(3): 693–705. doi: 10.1017/S0025315415000764

[B114] Townsend CH (1916) Voyage of the ‘Albatross’ to the Gulf of California in 1911. Bulletin of the American Museum of Natural History 35(24): 399–476. http://hdl.handle.net/2246/1019

[B115] Treadwell AL (1902) The polychaetous annelids of Porto Rico. Bulletin of the United States Fish Commission. 20, 2: 181–210. http://www.marinespecies.org/polychaeta/aphia.php?p=sourcedetails&id=126990

[B116] Treadwell AL (1929) New species of polychaetous annelids in the collections of the American Museum of Natural History from Porto Rico, Florida, Lower California, and British Somaliland. American Museum Novitates 392: 1–13. http://hdl.handle.net/2246/3789

[B117] Treadwell AL (1931) Three new species of polychaetous annelids in the collections of the United States National Museum. Proceedings of the U.S. National Museum 80(2902): 1–5. http://biodiversitylibrary.org/page/7614489

[B118] Treadwell AL (1939) Polychaetous Annelids of Porto Rico and Vicinity. New York Academy of Sciences. Scientific Survey of Porto Rico and the Virgin Islands 16(2): 151–319. https://archive.org/details/scientificsurvey160104newy

[B119] Verrill AE (1873) VIII. Report upon the invertebrate animals of Vineyard Sound and the adjacent waters, with an account of the physical characters of the region. Report of the United States Commission of Fish and Fisheries, 757 pp. doi: 10.5962/bhl.title.11688

[B120] Welter-Schultes FW (2013) Guidelines for the Capture and Management of Digital Zoological Names Information. Version 1.1, [Version 1.0 released 2012] 126 pp. http://www.gbif.org/orc/?doc_id=2784

[B121] Wu BL Chen M (1981) Two new species of *Hydroides* (Polychaeta: Serpulidae) from South China Sea. Oceanologia et Limnologia Sinica 12(4): 354–357.

[B122] Zibrowius H (1968) Étude morphologique, systématique et écologique des Serpulidae (Annelida Polychaeta) de la région de Marseille. Recueil des Travaux de la Station Marine d’Endoume 43(59): 81–252.

[B123] Zibrowius H (1971) Les espèces Méditerranéennes du genre *Hydroides* (Polychaeta Serpulidae). Remarques sur le pretendu polymorphisme de *Hydroides uncinata* Tethys 2: 691–746. http://paleopolis.rediris.es/benthos/REF/som/T-pdf/1970_2-3-691.pdf

[B124] Zibrowius H (1972a) Deux espèces nouvelles du genre *Hydroides* (Polychaeta, Serpulidae) de la Mer Jaune et des Iles Banda. Bulletin de la Société Zoologique de France 97(1): 89–93.

[B125] Zibrowius H (1972b) *Hydroides norvegica* Gunnerus, *Hydroides azorica* n.sp. et *Hydroides capensis* n. sp. (Polychaeta Serpulidae) espèces vicariantes dans l’Atlantique. Bulletin du Muséum d’Histoire Naturelle, Paris, Series 3, 33(39): 433–446.

[B126] Zibrowius H (1972c) Mise au point sur les espèces Méditeranéennes de Serpulidae (Annelida Polychaeta) décrites par Stefano delle Chiaje (1822–1829, 1841–1844) et Oronzio Gabriele Costa (1861). Tethys 4(1): 113–126. http://paleopolis.rediris.es/benthos/REF/som/T-pdf/1972_4-1-113.pdf

[B127] Zibrowius H (1973) Serpulidae (Annelida Polychaeta) des côtes ouest de l’Afrique et des archipels voisins. Annales Musee Royal de l’Afrique Centrale, Ser. 8, Sciences Zoologique 207: 1–93.

[B128] Zibrowius H (1978) Introduction du polychète Serpulidae Japonais *Hydroides ezoensis* sur la côte Atlantique Française et remarques sur la propagation d’autres espèces de Serpulidae Tethys 8(2): 141–150. http://paleopolis.rediris.es/benthos/REF/som/T-pdf/1976_8-2-141.pdf

[B129] Zoological Museum, Ivan Franko National University of Lviv (undated) Benedykt Dybowski (12.V.1833–31.I.1930). http://zoomus.lviv.ua/en/dybovsky/ [Accessed on 28 July 2016]

